# Non-cell autonomous regulation of cell–cell signaling and differentiation by mitochondrial ROS

**DOI:** 10.1083/jcb.202401084

**Published:** 2024-11-13

**Authors:** Yipeng Du, Lei Wang, Lizbeth Perez-Castro, Maralice Conacci-Sorrell, Matthew Sieber

**Affiliations:** 1 Department of Physiology, UT Southwestern Medical Center, Dallas, TX, USA; 2 Department of Cell Biology, UT Southwestern Medical Center, Dallas, TX, USA

## Abstract

Mitochondrial reactive oxygen species (ROS) function intrinsically within cells to induce cell damage, regulate transcription, and cause genome instability. However, we know little about how mitochondrial ROS production non-cell autonomously impacts cell–cell signaling. Here, we show that mitochondrial dysfunction inhibits the plasma membrane localization of cell surface receptors that drive cell–cell communication during oogenesis. Within minutes, we found that mitochondrial ROS impairs exocyst membrane binding and leads to defective endosomal recycling. This endosomal defect impairs the trafficking of receptors, such as the Notch ligand Delta, during oogenesis. Remarkably, we found that overexpressing RAB11 restores ligand trafficking and rescues the developmental defects caused by ROS production. ROS production from adjacent cells acutely initiates a transcriptional response associated with growth and migration by suppressing Notch signaling and inducing extra cellualr matrix (ECM) remodeling. Our work reveals a conserved rapid response to ROS production that links mitochondrial dysfunction to the non-cell autonomous regulation of cell–cell signaling.

## Teaser

Mitochondrial reactive oxygen species production is a major driver of aging and disease. Du et al. report that mitochondrial ROS production disrupts cell-cell communication by disrupting membrane receptor trafficking. Restoring endosomal trafficking of these receptors improves cell-cell signaling and rescues normal development.

## Introduction

Mitochondrial metabolism is a significant factor in disease progression and aging (Gorman et al., 2016; Lima et al., 2022). Defects in mitochondrial metabolism are major contributing factors in diseases such as Alzheimer’s, Parkinson’s, cardiovascular disease, metabolic syndrome, and cancer (Zhou and Tian, 2018; Vyas et al., 2016; Wang et al., 2020; Forman and Zhang, 2021). Most studies of mitochondrial dysfunction examine its intrinsic impact on redox metabolism, calcium signaling (Cao and Chen, 2009), apoptosis (Tait and Green, 2012; Zong et al., 2016), and cellular damage via reactive oxygen species (ROS) (Schieber and Chandel, 2014; Alfadda and Sallam, 2012). ROS is thought to mediate its effects on biology by altering transcription, causing oxidative damage to lipids and proteins, and inducing DNA damage. While it has been shown that ROS can impact developmental cell signaling pathways (Sinenko et al., 2011; Owusu-Ansah and Banerjee, 2009, Zhang et al., 2013; Rendra et al., 2019) in many cases, the mechanism linking ROS production to defects in developmental signaling remains unclear.

Most studies examining the role of ROS in developmental signaling examine the intrinsic effect of high ROS levels on the signaling responses within the cell (Zhang et al., 2013; Owusu-Ansah and Banerjee, 2009; Sinenko et al., 2011; Poplawski et al., 2019; Chen et al., 2023). ROS are charged molecules that cannot cross lipid membranes directly (Sies and Jones, 2020). Instead, ROS can escape cells through specific subsets of plasma membrane channels such as Aquaporins (Hirt, 2016; Tamma et al., 2018). Recent studies in central nervous system (CNS) suggest that lipid oxidation and lipid transport are essential contributors to the non-cell autonomous aspects of ROS in neural degeneration and oxidative stress (Liu et al., 2015; Ioannou et al., 2019; Haynes et al., 2024). However, very little is known about how mitochondrial dysfunction and ROS production impact broader aspects of global protein trafficking and cell–cell communication. As a result, surprisingly little is known about how the corruption of mitochondrial metabolism in one cell, non-cell autonomously, regulates cellular identity and signaling in adjacent cells.

Using a combination of model system genetics and human cell culture systems, we have found that mitochondrial metabolism can regulate differentiation non-cell autonomously in vivo. We have found that inducing ROS production in germ cells blocks the Delta/Notch-mediated differentiation of adjacent follicle cells (FCs). ROS controls Notch signaling by regulating endosomal recycling and the membrane localization of the ligand Delta. Using human cell culture models, we have found that ROS production plays a conserved role in regulating protein trafficking and secretion by controlling endosome recycling. We have found that ROS production influences endosome recycling by controlling the membrane-targeting complex, Exocyst, binding to the plasma membrane. This ROS response mechanism prevents the trafficking of cell surface ligands, such as Delta, from reaching the plasma membrane. Using human co-culture models, we have found that ROS production from adjacent cells induces a pro-metastatic/cell migration gene expression program that suppresses cell–cell signaling responses (Notch and Innate immune signaling) while promoting ECM remodeling. Our work defines an unappreciated role of ROS production in regulating endosomal recycling that drives the non-cell-autonomous roles for mitochondria in multiple systems.

## Results

### Germline mitochondrial dysfunction prevents FC development

**Figure 1.**
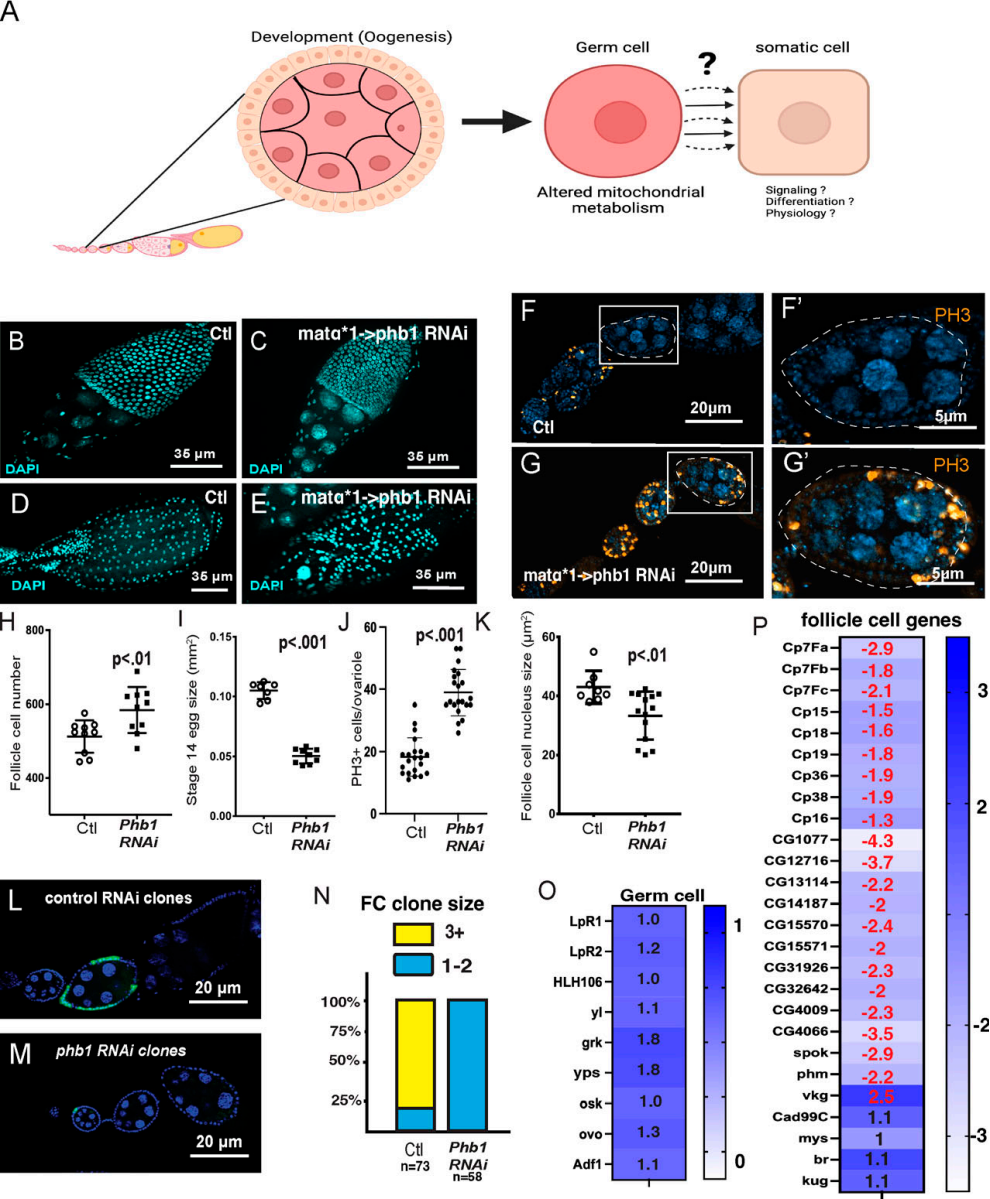
**Germ cell Mitochondrial dysfunction prevents follicle cell differentiation. (A)** A diagram describing the use of Drosophila oogenesis to examine the non-cell autonomous effects of germline mitochondrial dysfunction on somatic follicle cell development. **(B–E)** DAPI stained images of stage 10 and stage 14 egg chambers from control and PHB1-RNAi ovaries. **(F and G)** Ovarioles stained with DAPI and phosphohistone H3 (red) from the matα–>DsRed-RNAi (control) and matα (one copy)–>PHB1-RNAi females. F′ and G′ are zoomed-in images of egg chambers from F and G. **(H)** Follicle cell number counts from stage 10 egg chambers represented as a box and whisker plot. (*n* = 25 follicles). **(I)** Size measurements of stage 14 egg chambers. The line in the plot represents the average value. *n* = 10 egg chambers. **(J)** The number of PH3+ follicle cells from control and matα–>PHB1-RNAi females (*n* = 30 ovarioles). **(K)** Follicle cell nucleus size measurements of control and PHB1-RNAi stage 10 egg chambers (*N* = 20). **(L and M)** Images of Control and PHB1-RNAi follicle cell clones: blue represents DAPI, and green represents GFP from the follicle cell clone. **(N)** A graph representing the frequency of one to two cell clones in control RNAi clones and PHB1-RNAi clones. **(O)** RNA-seq data examining the expression of known germ cell-expressed genes. The precise fold change is overlayed on the heatmap. (Genes marked with a red fold change display an FDR < 0.05). *LpR1, LpR2,* and *yl* are known to be expressed in germ cells during late oogenesis (Stage 9–12). P) RNA-seq data examining the expression of known follicle cell-expressed genes (genes marked in red display an FDR < 0.05). The control used in all RNAi experiments is mata ->dsRED-RNAi All RNA seq-data represents three biological replicates. Student’s *t* test was used for all pairwise comparisons, and one-way ANOVA was used for all experiments containing >2 sample groups. Error bars represent the standard deviation.

*Drosophila* oogenesis provides a simplified system where two cell types, germ cells, and somatic FCs, develop in a highly coordinated manner (Fig. 1 A). Moreover, oogenesis provides the genetic tools and a system with well-defined cell–cell signaling relationships to examine the role of mitochondrial dysfunction in cell–cell signaling. Previous studies have shown that disruptions in mitochondrial metabolism cause defects in early germ cell development in the germarium (Teixeira et al., 2015). To avoid these early germarium defects and examine later phenotypes associated with mitochondrial disruption, we used the well-characterized germ line-specific driver matα-GAL4 (Hudson and Cooley, 2014), to inhibit the expression of mitochondrial genes in germ cells. From candidate-based genetic screens of mitochondrial genes, we identified *Phb1* (also called *l(2)37Cc*) as a mitochondrial factor that caused distinct phenotypes in germ cells and FCs when inhibited in the germline. *Phb1* is a mitochondrial chaperone/scaffold that forms a ring-like structure in the inner mitochondrial membrane with its binding partner Phb2 (Tatsuta et al., 2005).

**Figure S1.**
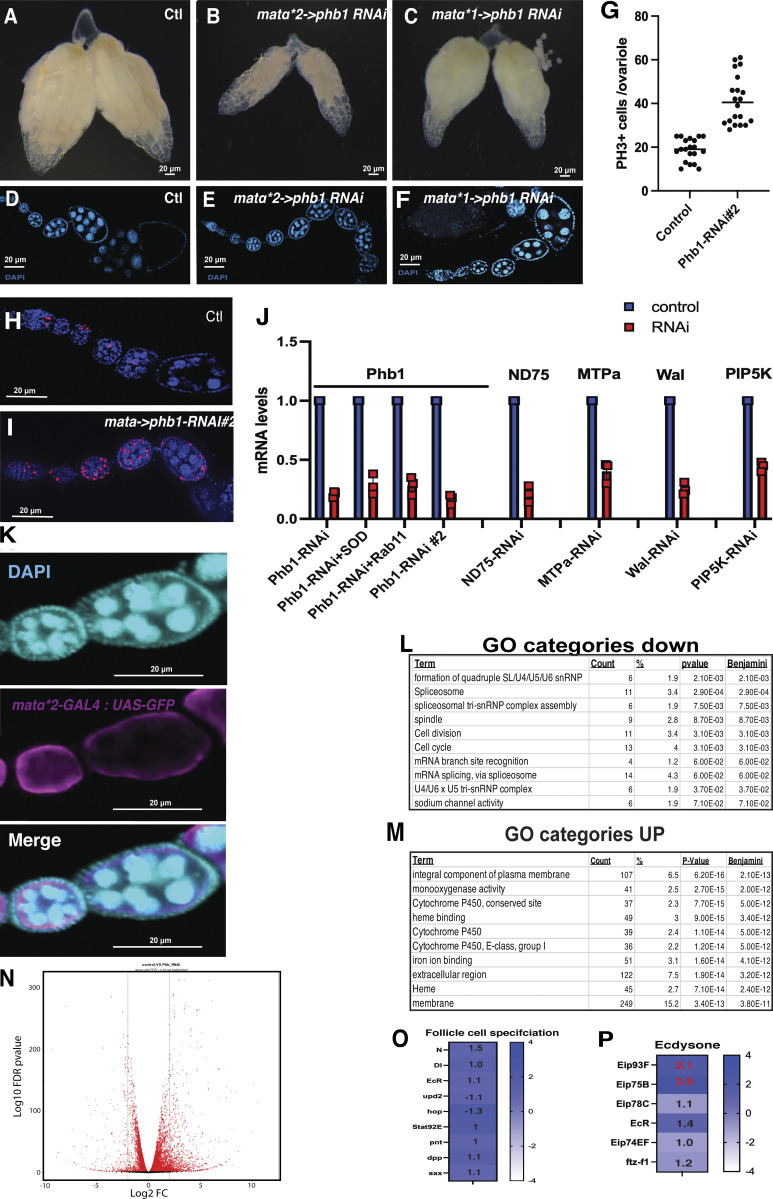
**Germline mitochondrial dysfunction disrupts Drosophila ooegenesis. (A–C)** Whole ovary images from control, mataX2(2 copies)–> *PHB-RNAi*, and mataX1*PhB-RNAi* females. **(D–F)** DAPI staining images of control, mataX2 *PhB-RNAi*, and mataX1*PhB-RNAi* ovarioles. **(G)** PH3+ positive cell counts from control and PHB1-RNAi#2 ovarioles (*n* = 20). **(H and I)** PH3 staining images of control and PHB1-RNAi #2 ovarioles. **(J)** RNAi-knockdown efficiencies measured by Q-PCR from control and RNAi transgenes used in this manuscript (*n* = 3). **(K)** GFP antibody staining images from the mata-GAL4-> UAS-GFP ovarioles confirming the germline-specific expression of the driver. **(L)** Gene ontology enrichment analysis for genes Downregulated in the PHB1-RNAi follicles. **(M)** Gene ontology enrichment analysis for genes upregulated in the PHB1-RNAi follicles. **(N)** A volcano plot of RNA-seq data comparing mRNA expression between control follicles and PHB1-RNAi expressing oocytes (FDR < 0.05). **(O)** RNA seq- data examining the expression of genes involved with follicle cell specification and patterning in PHB1-RNAi follicles. **(P)** RNA-seq data examining the expression of ecdysone pathway genes in PHB-RNAI follicles. (Genes marked with a red fold change display an FDR < 0.05). The control used in all RNAi experiments is mata ->dsRED-RNAi. Student’s *t* test was used for all pairwise comparisons, and one-way ANOVA was used for all experiments containing >2 sample groups. Error bars represent the standard deviation.

To examine the function of *Phb1* in germ cells, we used two copies of matα-GAL4 to induce potent RNAi silencing and one copy of matα-GAL4 to induce intermediate silencing. Interestingly, potent silencing of *Phb1* using two copies of matα-GAL4 to drive *Phb1-RNAi* expression caused a small ovary phenotype, triggered death during stage 8 of oogenesis, and arrested egg chamber development during vitellogenesis (yolk accumulation) (Fig. S1, A and B). This delay led to an accumulation of early egg chambers with strong Phb1-silencing (Fig. S1, D and E). Using one copy of matα-GAL4 to drive intermediate silencing of *Phb1-RNAi* reduced *Phb1* mRNA levels by roughly 75–80% (Fig. S1 J) and had no significant impact on egg laying (112.3 ± 18.4 versus 97.8 ± 13.7) and no significant effect on egg chamber development (Fig. S1, C and F). We focused our subsequent studies on this model with one copy of matα-GAL4 to avoid secondary effects caused by defective germ cell development. Moreover, we confirmed that this well-characterized matα-GAL4 driver is not expressed in the FCs (Fig. S1 K). Using this system, we examined how moderate silencing of *Phb1* expression in germ cells may impact the development of adjacent FCs.

Interestingly, moderate inhibition of *Phb1* expression caused an increased FC number (Fig. 1, B, C, and H) and disorganization of the FC epithelia in 34% of follicles (Fig. 1, D and E). The disorganization was most pronounced in stage 14 follicles. We also observed fused dorsal appendages (26%) and short/thick dorsal appendages in 32% of egg chambers. These phenotypes are commonly observed in egg chambers with defective FC specification (Berg, 2005), suggesting that FC specification and differentiation are impaired in *Phb1-RNAi* ovarioles.

These ovaries also display a 50% reduction in stage 14 egg chamber size (Fig. 1 I), a phenotype observed when FC patency is disrupted (Row et al., 2021). Patency is defined as the opening of intracellular spaces between cells and is known to be caused by defects in FC differentiation (Row et al., 2021). This stage 14 egg chamber size reduction likely accounts for the size reduction seen in matα-*Phb1-RNAi* ovaries. We observed an increased FC number in stage 10 *Phb1-RNAi* egg chambers (Fig. 1 H), suggesting that FCs continue proliferating and fail to enter the endocycle during mid-oogenesis. To determine if inhibiting Phb1-expression in germ cells non-cell autonomously regulates FC proliferation, we measured the number of mitotic cells (PH3+) in *Phb1-RNAi* ovarioles. Silencing *Phb1* causes significantly more proliferation in *Phb1-RNAi* ovarioles than in controls (Fig. 1, F–G′ and J). We confirmed this over-proliferation phenotype with a second PHB1-RNAi transgene (PHB1-RNAi#2) (Fig. S1, G–I). Moreover, FCs display reduced nuclear size in matα-*Phb1-RNAi* ovarioles (one copy of Gal4) (Fig. 1 K), consistent with the model where *Phb1* is required in germ cells to facilitate the mitosis to endocycle transition during FC development (Ward et al., 2006). Next, we examined *Phb1-RNAi* in FCs, which yielded different phenotypes than those we observed in Our germline RNAi experiments. When we induce GFP-marked *Phb1-RNAi* clones in the FC epithelia using a hs-Flp; Act>stop>Gal4; UAS-GFP line, we find disrupting *Phb1* directly in FCs yields small one to two cell clones 3 days post heat shock (1.4 cells average clone size). In contrast, control DsRed-RNAi clones grow much larger during the 3 days (14.3 average clone size) (Fig. 1, L–N). These data indicate that disrupting *Phb1* in the follicle cells affects cell growth rather than FC differentiation. These data further support that germline *Phb1* is required non-cell autonomously for FC differentiation.

Given that the M/E transition is part of the FC differentiation process (Ward et al., 2006; Sun et al., 2008), we wanted to systematically assess the expression of germ cell and FC genes in control and matαGAL4*1-*Phb1-RNAi* ovarioles. We isolated ovarioles from control and *Phb1-RNAi* females, purified the mRNA, and subjected it to RNA sequencing. From this analysis, we identified 2,861 genes miss-regulated at least twofold in *Phb1-RNAi* follicle (FDR < 0.05) (Fig. S1 N and Table S1). Over two-thirds of those genes are upregulated. These data are consistent with elevated stress levels in ovarioles where Phb1-Expression has been silenced by RNAi. When we examined the gene ontology terms associated with genes downregulated in *Phb1-RNAi* ovarioles, we observed a variety of terms weakly enriched in our data sets, such as snRNP, spliceosome, spindle, cell division, and sodium channel activity (Fig. S1 L). Examining the gene ontology terms associated with genes upregulated in *Phb1-RNAi* ovarioles found that terms such as plasma membrane component, monooxygenase, Cytochrome p450 (mitochondrial and ER-localized), and extracellular region were all significantly enriched in these data (Fig. S1 M). We examined the expression of well-characterized germ cell-expressed genes (grk, yps, osk, ovo, and Adf1) and found no defects in their expression (Fig. 1 O). We also looked at the expression of genes expressed in vitellogenic stage (yolk accumulating) germ cells (LpR2, LpR1, HLH106, and yl) (Fig. 1 O). We found no defects in the expression consistent with the normal germline development observed in *Phb1-RNAi* egg chambers (one copy of driver).

In contrast, when we examine the mRNA levels of the known chorion proteins, a family of genes expressed specifically in late-stage FCs (beginning in stage 10), we find all 19 chorion genes downregulated upon Phb1-silencing (Fig. 1 P). We also observed downregulation of the ecdysone biosynthetic genes spook and phantom a pair of steroid-producing cytochrome p450’s expressed explicitly in mature FCs in late-stage egg chambers (Fig. 1 P). Interestingly, genes expressed in egg chambers earlier during oogenesis (expression prior to M/E transition), such as vkg, mys, Cad99C, br, and Kug, are not downregulated in *Phb1-RNAi* ovarioles (Fig. 1 P). These data suggest an uncoupling of germline and FC development where germ cells continue to develop. In contrast, FCs fail to enter the endocycle and properly differentiate during late oogenesis. When we examine the expression of the ecdysone pathway and other known regulators of FC specification such as gurken, Notch, Delta, unpaired2, hop, stat92E, dpp, and saxophone, we observed no downregulation of any critical factors involved in FC specification (Fig. S1, O and P). These data suggest that the observed defects in FC development are not likely due to a transcriptional downregulation of known follicle cell regulating pathways.

### Germline mitochondrial dysfunction prevents Notch-mediated differentiation of FCs

**Figure 2.**
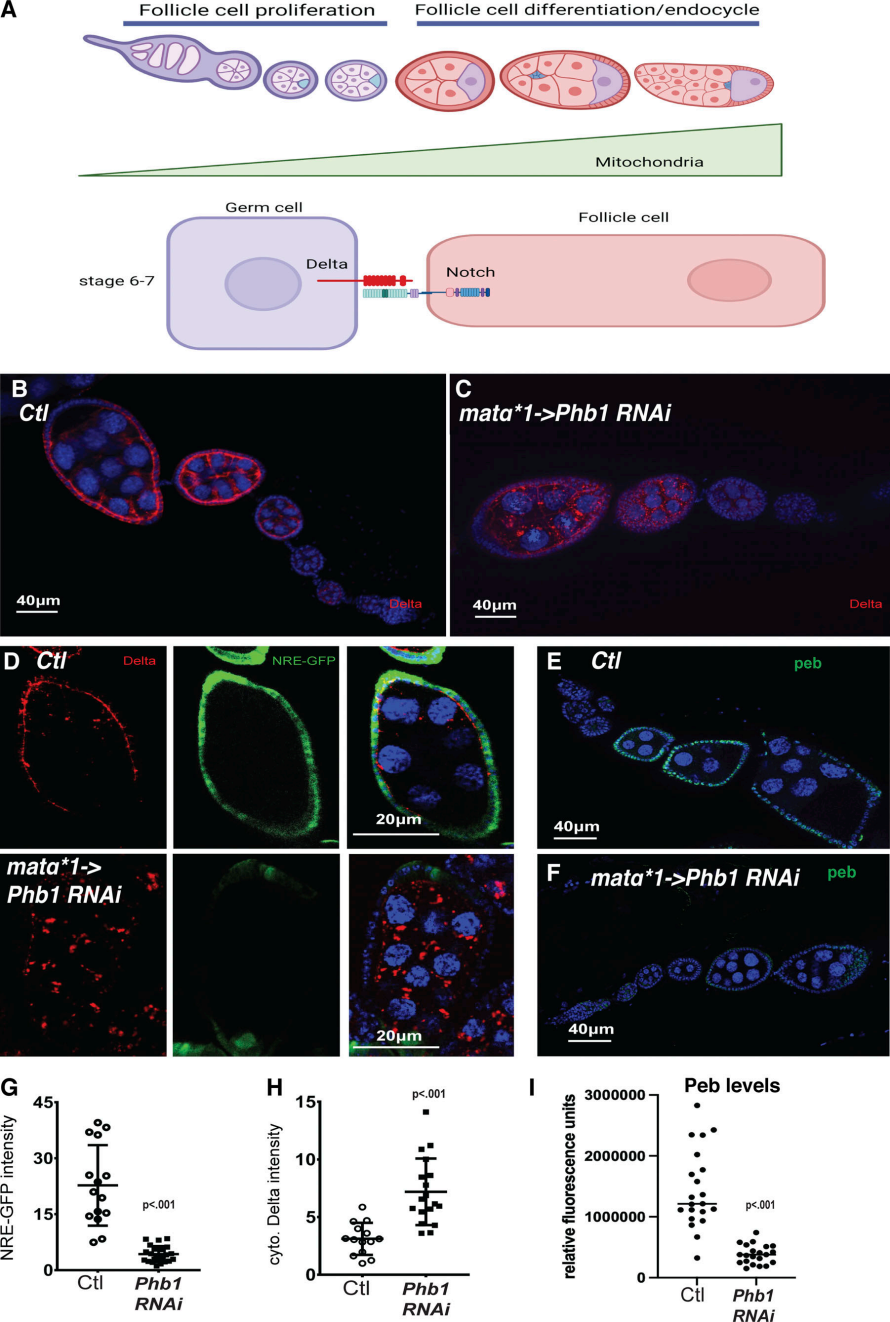
**Mitochondrial dysfunction impairs Notch signaling between germ cells and follicle cells. (A)** A diagram depicting the mitosis to endocycle transition, a very early step in follicle cell differentiation, and its regulation by Notch signaling. **(B and C)** Ovarioles from dsRED-RNAi control and PHB1-RNAi females stained with Delta ligand antibodies (red) and DAPI. **(D)** Immunofluorescence images of control and PHB1-RNAi egg chambers stained with Delta Antibodies (Red), NRE-GFP reporter(green), and DAPI. **(E and F)** immunofluorescence images of dsRED-RNAi control and PHB1-RNAi ovarioles stained with peb antibodies (green) and DAPI. **(G)** A graph showing NRE-GFP expression in control and PHB1-RNAi egg chambers (*n* = 20 follicles). **(H)** A graph showing the differences in cytosolic delta levels (*n* = 20 follicles). **(I)** Peb expression levels in control and Phb1-RNAi ovarioles (*n* = 20). Student’s *t* test was used for all pairwise comparisons, and one-way ANOVA was used for all experiments containing >2 sample groups. Error bars represent the standard deviation.

**Figure S2.**
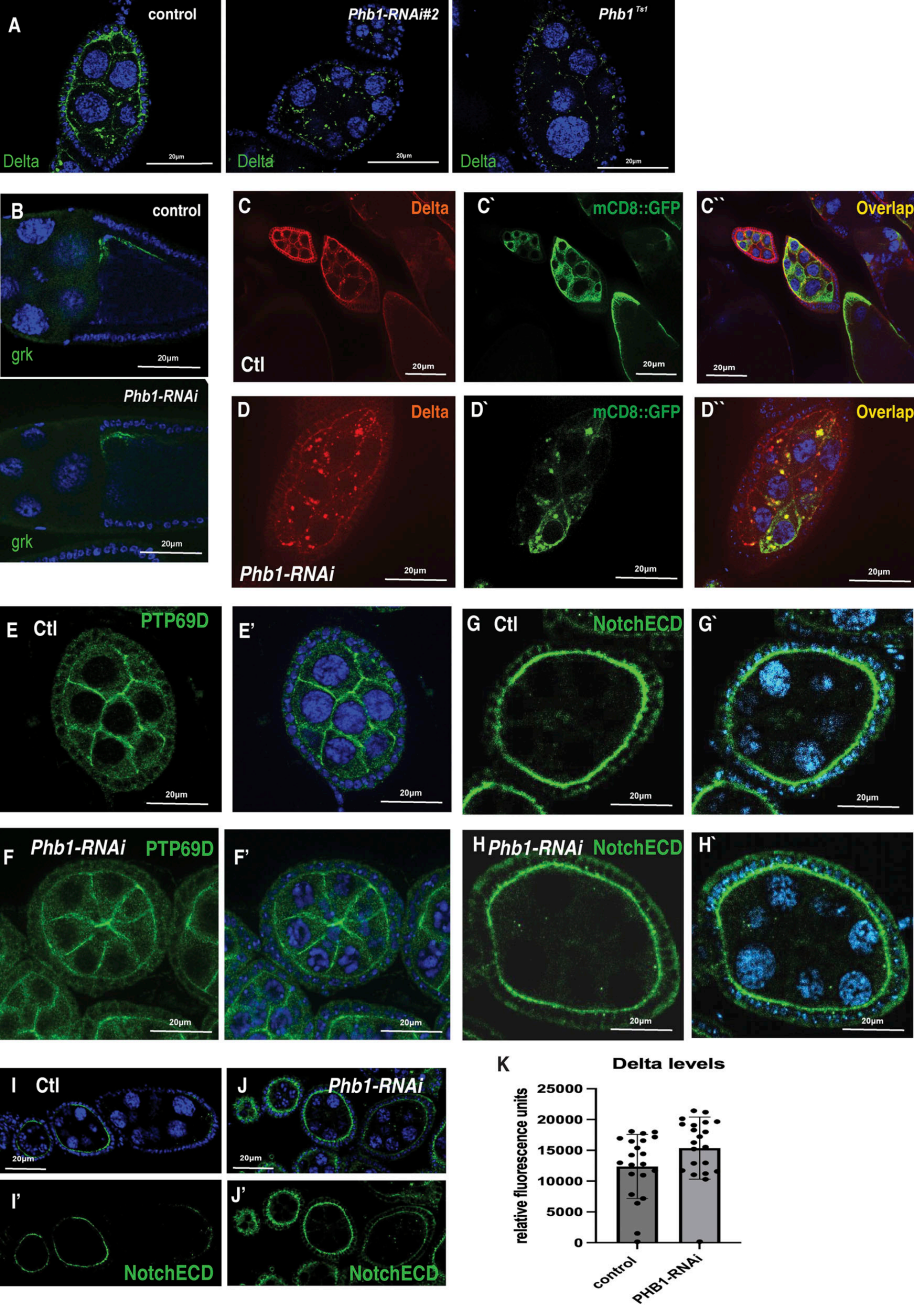
**Mitochondrial dysfunction imairs the trafficking to specific receptors. (A)** Images of egg chambers from control, PHB1-RNAI #2, and *PHB1*^*TS1*^ temperature-sensitive mutants stained for Delta ligand green. **(B)** Gurken antibody-stained egg chambers from control and PHB1-RNAi females. **(C–D’’)** Immunostaining of control and PHB1-RNAi egg chambers depicting delta (red) localization and mCD8-GFP (green) localization. **(E–F′)** Images of PTP69D staining in control and PHB1-RNAi ovarioles. **(G–J)** Notch-ECD (green) staining images of control and PHB1-RNAi egg chambers. **(K)** Fluorescence levels from antibody-stained images of control and PHB1-RNAi ovarioles (*n* = 25). Student’s *t* test was used for all pairwise comparisons, and one-way ANOVA was used for all experiments containing >2 sample groups. Error bars represent the standard deviation.

**Figure S3.**
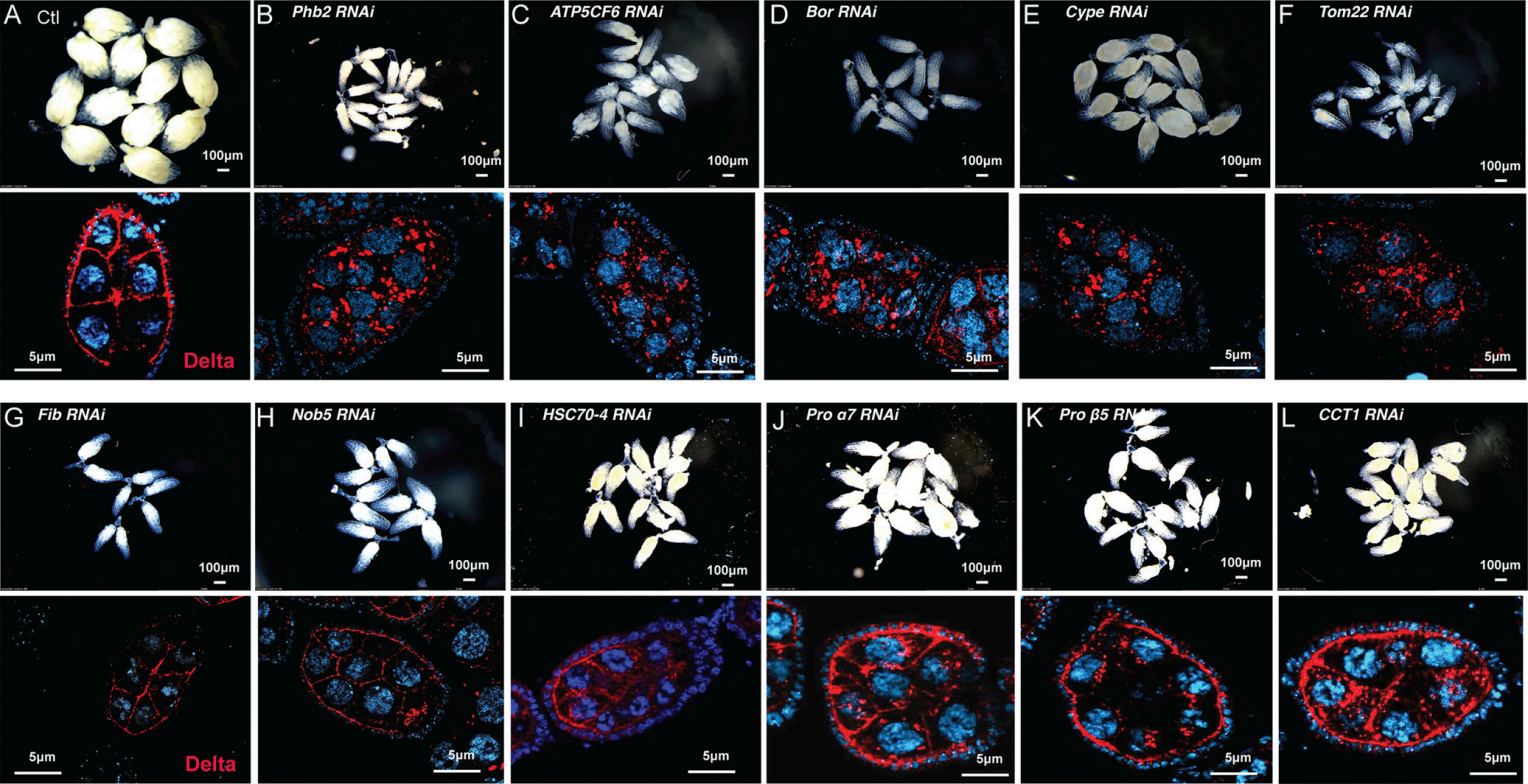
**Mitochondrial dysfunction prevents Delta trafficking independent of developmental arrest. (A–F)** Whole ovariole images and images of Delta stained ovarioles from RNAi transgenes for known mitochondrial genes, including control, PHB2-RNAi, ATP5CF6-RNAi, Bor-RNAi, Cype-RNAi, and tomm22-RNAi. Whole ovary images represent females with two copies of the matα-GAL4 driver and immunofluorescence images ovarioles from females carrying one-Copy of the matα-GAL4 driver. **(G–L)** Whole ovariole images and images of Delta stained ovarioles from RNAi transgenes for non-mitochondrial genes including Fib-RNAi, Nob5-RNAi, HSC-70-4-RNAi, Prosalpha7-RNAi, Prosbeta5-RNAi, and OCT1-RNAi. Whole ovary images represent females with two copies of the matα-GAL4 driver and immunofluorescence images ovarioles from females carrying one-Copy of the matα-GAL4 driver.

Notch ligand Delta (Dl) is expressed during this transition in germ cells. It activates the Notch signaling pathway in FCs, causing FCs to stop proliferating and enter the endocycle (Fig. 2 A) (Shcherbata et al., 2004; Assa-Kunik et al., 2007; Sun et al., 2008). We examined Delta ligand expression and localization in control and matα-*Phb1-RNAi* (one copy of driver) ovarioles. We found Delta fails to localize to the plasma membrane in nurse cells (Fig. 2, B–D). We confirmed these results using a temperature-sensitive allele of *Phb1* (*Phb1*^*TS1*^). We found that when grown at the non-permissive temperature, *Phb1*^*TS1*^ egg chambers do not have Delta on their nurse cell plasma membranes (Fig. S2 A). We also confirmed these results by inhibiting the expression of *Phb1* using a second RNAi line (Fig. S2 A) and by silencing the *Phb1* binding partner *Phb2* (Fig. S3 B) in germ cells and observing very similar phenotypes.

Moreover, the Notch signaling reporter NRE-GFP fails to activate in PHB1-RNAi ovarioles (Fig. 2, D and G). Delta appears to localize to cytosolic puncta in *Phb1-RNAi* egg chambers (Fig. 2, D and H). We confirmed these data by examining *pebble/peb* expression (*peb* is induced by Notch signaling during mid-oogenesis). We found *peb* expression was reduced in FCs from *Phb1-RNAi* egg chambers, indicating that notch signaling is defective in *Phb1-RNAi* egg chambers (Fig. 2, E, F, and I). These data suggest that Delta/Notch signaling is impaired in *Phb1-RNAi* egg chambers.

Interestingly, the FC specification regulator (Gurken) (Fig. S2 B) and other transmembrane proteins expressed in germ cells, such as PTP69D, display normal localization (Fig. S2, E–F′) in *Phb1-RNAi* ovarioles. These data suggest that global membrane trafficking is not impaired in *Phb1-RNAi* silencing. Membrane proteins such as CD8-GFP, however, do display defects in membrane trafficking when *Phb1-RNAi* is silenced in germ cells (Fig. S2, C and D). Delta and CD8-GFP show some overlap in the cytosolic puncta formed when *Phb1* is silenced. These data suggest that specific proteins, including Delta and CD8-GFP, require *Phb1* function in the mitochondria for normal trafficking.

We assayed Delta protein turnover in germ cells by examining the Notch extracellular domain (Notch-ECD). Delta turnover is directly coupled to Notch-ECD turnover in adjacent cells. (Coumailleau et al., 2009; Kopan, 2012). We postulated that defects in Delta turnover would also impact the levels of Notch-ECD, just as we reported in our previous work (Obniski et al., 2018). However, we do not observe consistent changes in Notch-ECD expression in *Phb1-RNAi* ovarioles relative to controls (Fig. S2, G–J). There was a delay in Notch-ECD downregulation, but the effects were highly variable (Fig. S2, G–J). This, in conjunction with the fact that we do not see any significant changes in the levels of Delta antibody staining fluorescence (Fig. S2 K), suggests that Delta turnover is unaffected in *Phb1-RNAi* ovarioles.

To exclude the possibility that developmental delays or arrests, like those we observed with strong *Phb1-RNAi* silencing, cause these delta trafficking defects, we examined five other mitochondrial genes identified in our candidate-based screen as well as six non-mitochondrial genes that cause small ovary and ovariole arrest phenotypes. Interestingly, silencing the mitochondrial genes (Phb2, ATP5CF6, Cype, and Tom22) caused Delta trafficking defects like what we observed in *Phb1-RNAi* egg chambers (Fig. S3, A–F). Silencing non-mitochondrial genes (Fib, Nob5, HSC70-4, Prosα7, Prosβ5, and CCT1) had no impact on Delta localization despite displaying gross developmental phenotypes like *Phb1-RNAi* egg chambers (Fig. S3, G–L). Based on these results, we hypothesized that a specific aspect of mitochondrial dysfunction in *Phb1-RNAi* egg chambers may be involved in Delta protein trafficking.

### Mitochondrial dysfunction controls FC differentiation via mitochondrial ROS production

**Figure 3.**
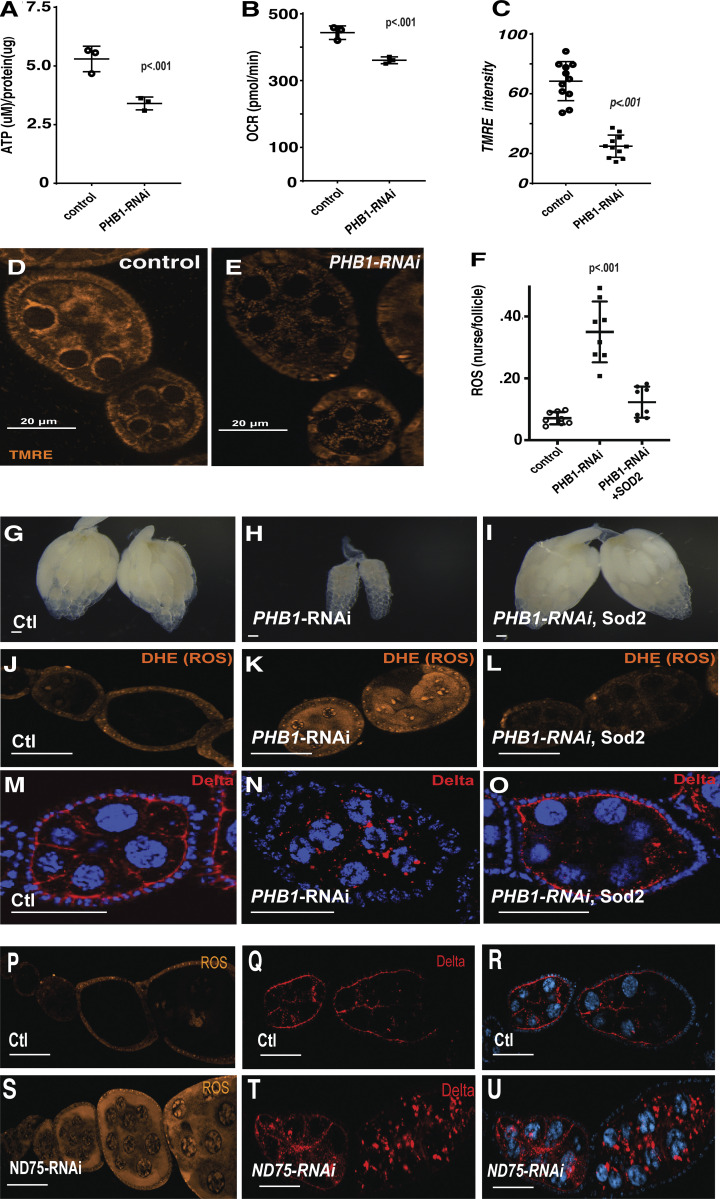
**ROS production regulates Delta ligand trafficking. (A)** ATP measurements from control and PHB1-RNAi egg chambers (*n* = 3 independent samples). **(B)** Seahorse-based oxygen consumption rate from control and PHB1-RNAi ovarioles (*n* = 6 sets of three ovarioles). **(C)** Measurements of TMRE fluorescence from control and PHB1-RNAi egg chambers (*n* = 10 independent samples). **(D and E)** TMRE stained images of control, PHB1-RNAi. **(F)** ROS measurements from control, PHB1-RNAi, and PHB1-RNAi +SOD2 ovarioles. Follicle cells function as an internal control. The data is expressed as a nurse cell/follicle cell fluorescence ratio. (*n* = 10 independent samples). **(G–I)** whole ovary images of control, PHB1-RNAi, and PHB1-RNAi +SOD2 egg chambers (2 copies of GAL4). **(J–L)** DHE staining images of ROS levels of control, PHB1-RNAi, and PHB1-RNAi +SOD2 egg chambers (1 copy of GAL4). **(M–O)** Immunofluorescence images of control, PHB1-RNAi, and PHB1-RNAi +SOD2 egg chambers (one copy of GAL4) stained with Delta antibodies. **(P and S)** DHE staining of control and ND-75-RNAi egg chambers. **(Q, R, T, and U)** Immunofluorescence images of control and ND-75-RNAi egg chambers stained with Delta antibodies (1 copy of GAL4). Student's *t* test was used for all pairwise comparisons, and one-way ANOVA was used for all experiments containing >2 sample groups. Error bars represent the standard deviation.

**Figure S4.**
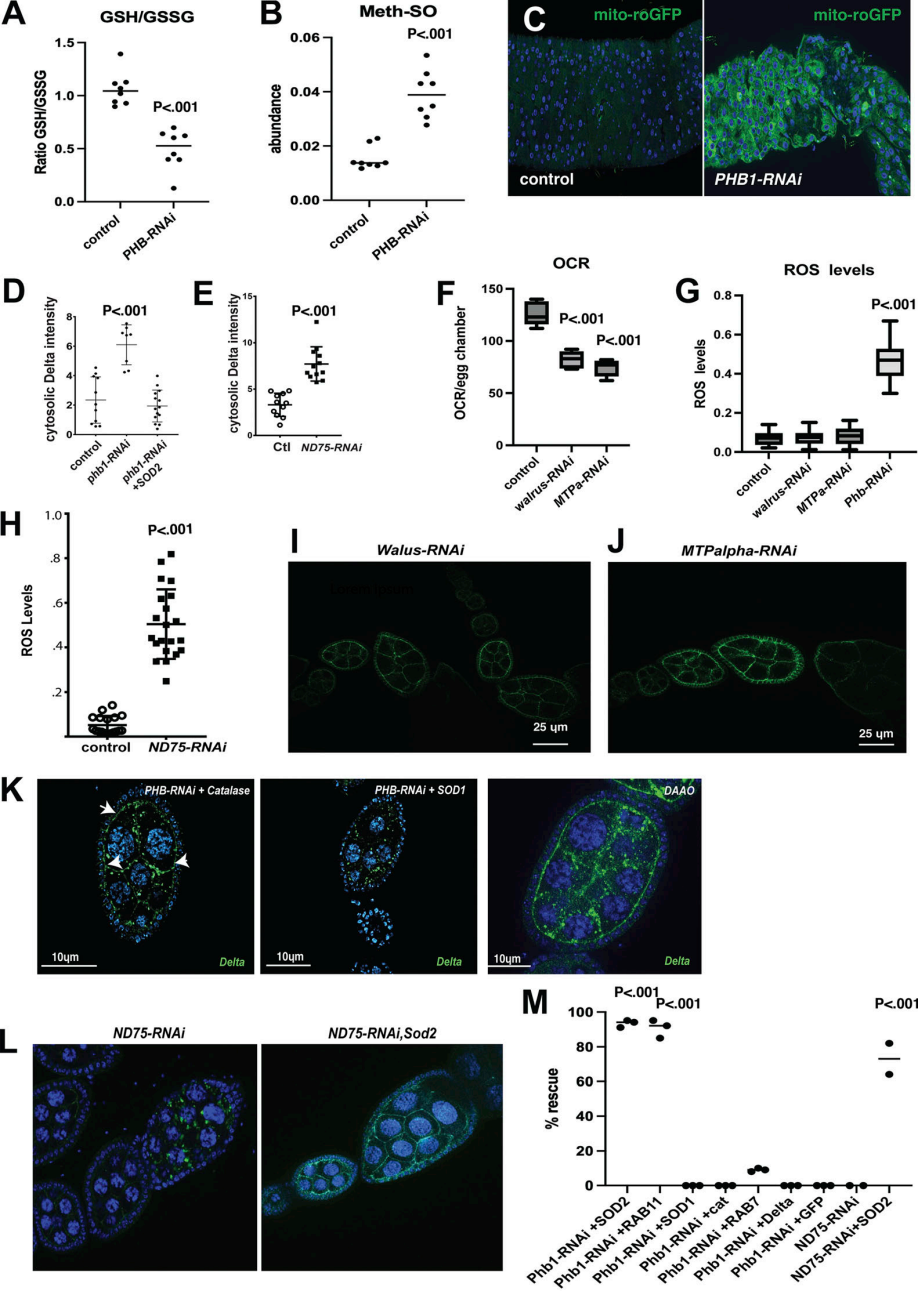
**ROS production regulates Delta protein trafficking. (A)** LC/MS GSH/GSSG ratio measurements from control and PHB1-RNAi egg chambers (*n* = 8 biological samples). **(B)** LC/MS measurements of Methionine-sulphoxide levels from control and PHB1-RNAi egg chambers (*n* = 8 biological samples). **(C)** images of mito-roGFP (green) fluorescence from control and PHB1-RNAi expression intestines. **(D)** Cytosolic delta levels of control, PHB1-RNAi, and PHB1-RNAi +SOD2 egg chambers (*n* = 20 independent samples). **(E)** Cytosolic delta levels of control and ND-75-RNAi egg chambers (*n* = 20 independent samples). **(F)** oxygen consumption rate measurements from control, walrus-RNAi, and MTPalpha-RNAi staged egg chambers (*N* = 6 sets stage 10 egg chambers). Egg chambers were used in this experiment to correct for delays in development. **(G)** ROS levels from control, walrus-RNAi, MTPalpha-RNAi, and ND75-RNAi egg chambers based on fluorescent imaging. Follicle cells are used as an internal control, and the data is expressed as a ratio of nurse cell/follicle cell fluorescence (*n* = 20 egg chambers). **(H)** ROS-level quantification from control and ND75-RNAi egg chambers (*n* = 20 egg chambers). **(I and J)** Delta staining images of ovarioles from Walrus-RNAI and MTPalpha-RNAi females. **(K)** Delta staining of PHB-RNAi+catalase, PHB-RNAi+SOD1, and DAAO-expressing ovarioles. Arrows point to areas where Delta localization to the membrane is rescued. **(L)** Delta staining images from ND75-RNAi and ND75-RNAi+SOD2 ovarioles. **(M)** A summary graph measuring the %rescue of ovarioles showing normal Delta membrane staining of all UAS-transgenes used in this study. The expression of all the transgenes used in this graph is driven by matα-GAL4. Each data point represents an experiment of at least 25 ovarioles. Student’s *t* test was used for all pairwise comparisons, and one-way ANOVA was used for all experiments containing >2 sample groups. Error bars represent the standard deviation.

We characterized mitochondrial function in control and *Phb1-RNAi* egg chambers. We found that *Phb1-RNAi* egg chambers display reduced mitochondrial respiration (oxygen consumption rate, OCR) and reduced levels of ATP relative to control egg chambers (Fig. 3, A and B). We also found that *Phb1-RNAi* egg chambers significantly reduced mitochondrial membrane potential (TMRE staining) (Fig. 3, C–E) relative to control consistent with proton leakage and reduced proton motive force using methods described in Sieber et al. (2016). We also found that *Phb1-RNAi* egg chambers display significant increases in ROS production when measured with the redox-sensitive stain DHE (Fig. 3, F, J, and K). However, fluorescent reporters/dyes used for ROS detection can be misleading due to concerns about autofluorescence, specificity, and overall sensitivity. As a result, we confirmed this increase in ROS by measuring glutathione ratios via LC/MS. Consistent with our staining results, we observed a significant reduction in the GSH/GSSG ratio (Fig. S4 A) in *Phb1-RNAi* egg chambers, indicating elevated levels of ROS production. We also measured the levels of oxidized methionine (methionine sulfoxide/Meth-SO) and found a threefold increase in methionine oxidation (Fig. S4 B). The enzyme methionine sulphoxide reductase, MSR, typically converts meth-SO back to methionine. However, the elevation of meth-SO in *Phb1-RNAi* egg chambers reflects sustained ROS production, which exceeds MSR’s capacity, caused by mitochondrial dysfunction in *Phb1-RNAi* egg chambers. We found that the available ROS reporters (roGFP) do not express well in *Drosophila* germ cells. As a result, we wanted to confirm that *Phb1-RNAi* silencing induces ROS production in *Drosophila* intestines. We confirmed that *Phb1-RNAi* causes ROS production in other tissues by silencing *Phb1* via RNAi in the intestinal epithelia and assayed for changes in Superoxide responsive roGFP-grx reporter fluorescence. Inhibiting *Phb1* in the intestine significantly increased roGFP reporter fluorescence, confirming that Phb1-silencing causes mitochondrial ROS production (Fig. S4 C).

To determine if ROS accumulation drives the observed developmental and Delta trafficking defects, we observed in *Phb1-RNAi* ovaries; we overexpressed mitochondrial SOD2 in *Phb1-RNAi* expressing germ cells. Interestingly, SOD2 expression completely rescued the developmental defects we observed in ovaries with strong *Phb1* silencing (matα*2-Phb1-RNAi) (Fig. 3, G–I). SOD2 expression also rescued the Delta trafficking defects in *Phb1-RNAi* egg chambers (Fig. 3, J–O; and Fig. S4, D and M). This rescue is not due to dilution of the GAL4 because UAS-SOD2 did not impact the knockdown efficiency of *Phb1* in our RNAi lines (Fig. S1 J). Moreover, several other UAS transgenes for various genes (Delta, GFP, Rab7, SOD1, etc.) did not rescue the phenotypes associated with Phb1 silencing.

Interestingly, previous scRNA-seq experiments (Li et al., 2022) show that germ cells express ∼10-fold higher levels of the H_2_O_2_ detoxifying enzyme gpx1 than somatic cells (germ cell = 2,242 versus somatic cell = 208 Flyatlas scRNA data) (Li et al., 2022). These data suggest that SOD2 levels in germ cells limit the clearance of mitochondrial ROS. These data indicate that ROS caused by mitochondrial dysfunction is the primary cause of delta trafficking defects we observed in *Phb1-RNAi* egg chambers. SOD1(cytosolic) overexpression did not rescue the Delta trafficking defects observed in *Phb1-RNAi* egg chambers, consistent with the fact that superoxide is being produced in the mitochondria of PHB1-RNAi oocytes (Fig. S4 K). However, catalase overexpression weakly rescues the Delta trafficking defects in *Phb1-RNAi* egg chambers (Fig. S4 K). This partial effect may reflect that germ cells express 10 times more of the H_2_O_2_ clearing enzyme, gpx1, than somatic cells (Li et al., 2022). Therefore, increasing catalase activity has a limited effect on H_2_O_2_ clearance and supports the idea that SOD2 levels are limiting for mitochondrial ROS clearance. However, it is possible that greater levels of catalase expression may yield a more complete rescue of *Phb1-RNAi* phenotypes.

*Phb1* is an mitochondrial chaperone that forms a ring-like structure in the inner mitochondrial membrane (Tatsuta et al., 2005), and its role in ROS production remains unclear. We confirmed this role of ROS in Delta trafficking by inducing ROS production using a UAS-DAAO construct that produces ROS without mitochondrial dysfunction and found that ROS produced by DAAO expression causes similar defects in Delta trafficking (Fig. S4 K).

We also confirmed the role of mitochondrial ROS production in Delta trafficking by inhibiting the mitochondrial ETC complex one subunit ND75(NDUFS1 in mammals). Targeting ND75 provides a tool to directly induce ROS production by disrupting complex one activity. In addition, reduced ND75/NDUFS1 levels are well known to cause ROS production in *Drosophila* and mammalian systems (Fig. S4 H) (Owusu-Ansah and Banerjee, 2009, Sinenko et al., 2011; Lopez-Fabuel et al., 2016; Zou et al., 2021; Chen et al., 2023). Inducing ROS production by complex 1 inhibition in germ cells by silencing ND75 fully replicates the Delta trafficking defects we observed in *Phb1-RNAi* egg chambers (Fig. 3, P–U and Fig. S4 E). Moreover, expressing SOD2 in ND75-RNAi flies rescues the Delta trafficking defects seen when ROS is produced (Fig. S4, L and M).

To confirm that ROS production, not impaired mitochondrial function, regulates Delta trafficking, we lowered mitochondrial respiration without inducing ROS production. We show that inhibiting the mitochondrial fatty acid oxidation pathway members Walrus (ETFa) and MTPα in germ cells reduced respiration (OCR) without changing ROS production in egg chambers (Fig. S4, F and G). Delta localization, however, was not affected by the silencing of walrus or MTPα (Fig. S4, I and J) further supporting the idea that ROS is the primary cause of Delta trafficking defects upon mitochondrial disruptions.

### High ROS levels prevent exocyst membrane association

**Figure 4.**
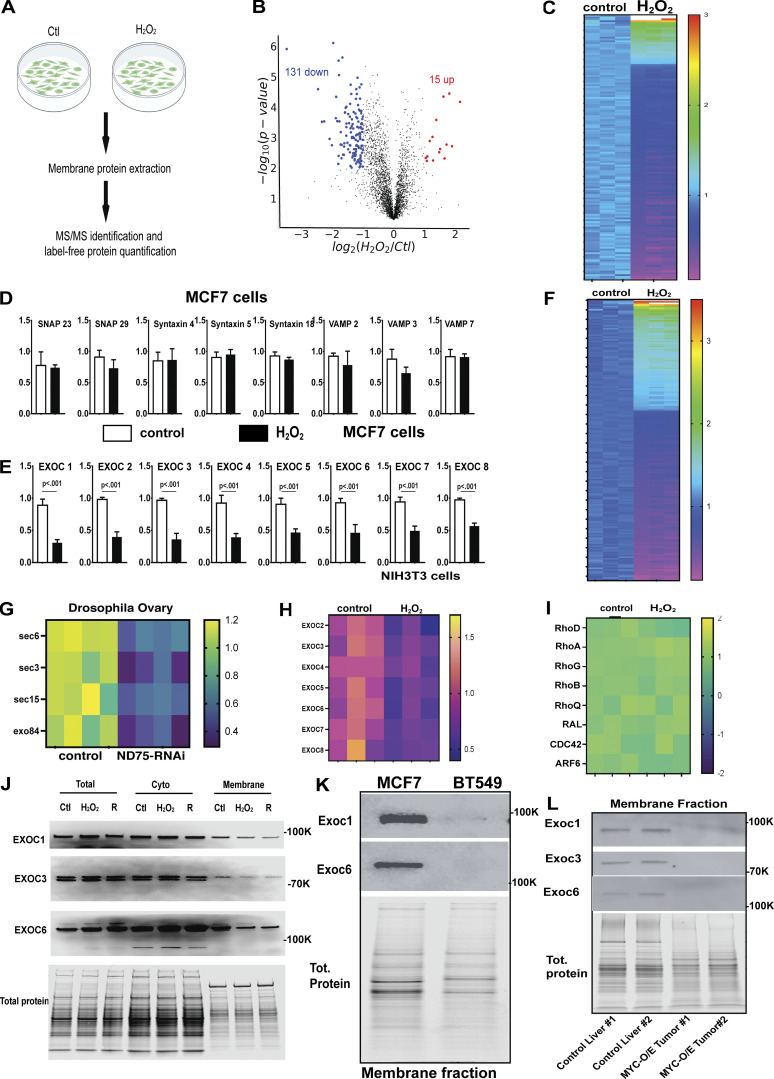
**ROS prevents exocyst binding to the membrane. (A)** A diagram describing the MCF7 cell model used to examine the impact of a 30-min ROS exposure on the membrane proteome. **(B)** A volcano plot examining the changes in membrane proteome caused by ROS exposure (FDR < 0.05). **(C)** A heat map depicting the expression changes of the top 100 most differentially regulated proteins in membranes isolated from ROS-exposed cells (FDR < 0.05). **(D)** Protein abundances for several common membrane proteins in control and H_2_O_2_-treated cells. **(E)** Membrane abundances for exocyst complex components in control and H_2_O_2_-treated MCF7 cells (FDR < 0.05). **(F)** A heat map showing the top 100 most differentially regulated proteins in membrane fraction from 3T3 cells and 3T3 treated with H_2_O_2_. **(G)** A heat map representing proteomic-based measurements of the detectable subunits of the exocyst complex from membrane fractions isolated from Drosophila eggs. **(H)** A heat map representing proteomic-based measurements of the detectable subunits of the exocyst complex from membrane fractions isolated from NIH3T3 cells. **(I)** A heat map representing proteomic-based measurements of known exocyst regulators from membrane fractions isolated from MCF7 cells. **(J)** Western blot validation for EXOC 1 and 6 from MFC7 total cell lysate, cytosolic fractions, and purified membrane fractions. **(K)** Western blots examining the levels of Exoc1 and Exoc6 in membrane fractions from MCF7 cells and BT549 cells. **(L)** Western blots examining Exoc1, Exoc3, and Exoc6 levels in control liver and Myc-O/E hepatic tumors. Student’s *t* test was used for all pairwise comparisons, and one-way ANOVA was used for all experiments containing >2 sample groups. Error bars represent the standard deviation. Source data are available for this figure: SourceData F4.

**Figure S5.**
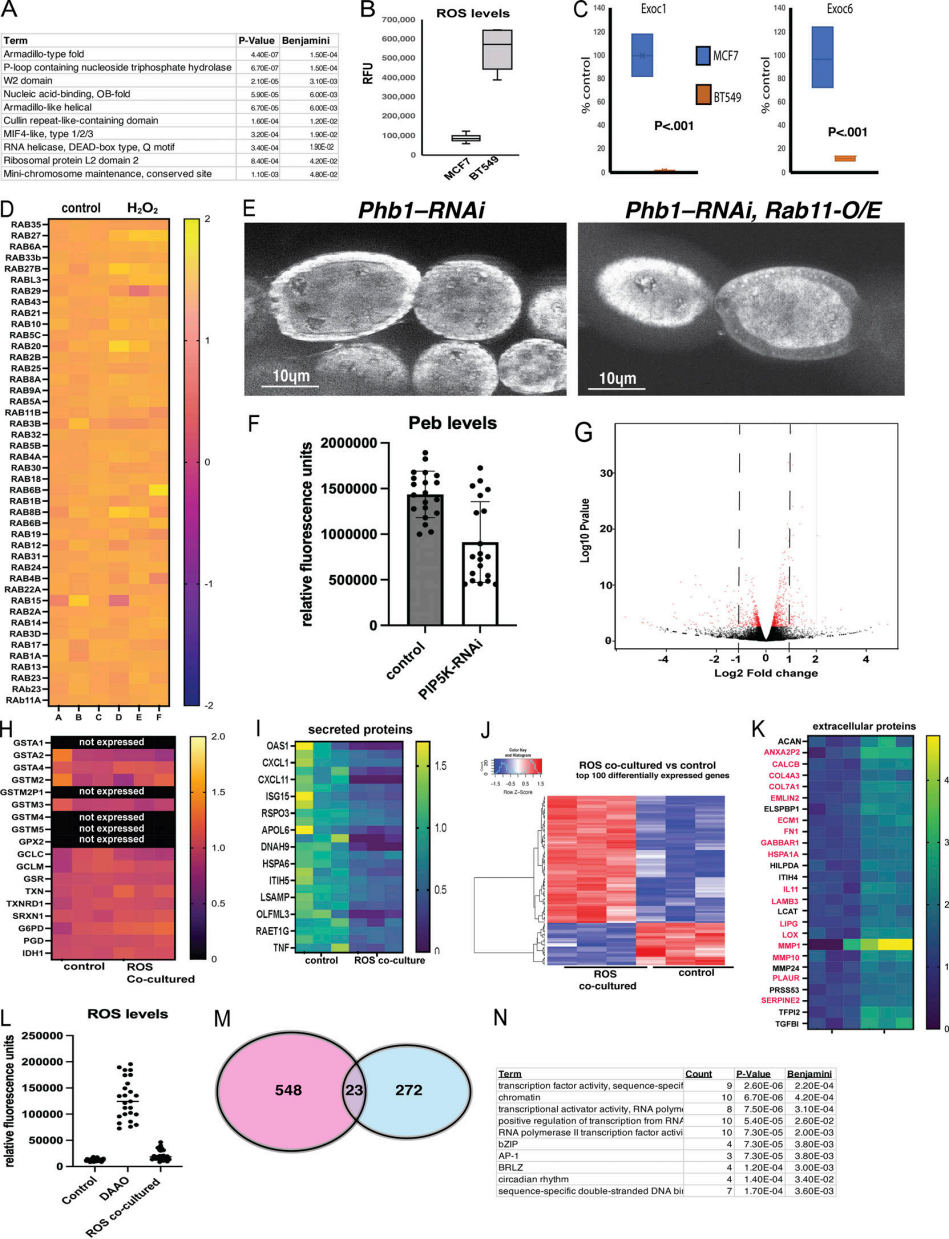
**ROS production disrupts endosomal traffikcing and cell cell communication. (A)** GO enrichment for protein domains found in proteins downregulated in membrane fractions purified from MCF7 cells exposed to ROS. **(B)** ROS levels from MCF7 cells and BT549 cells. **(C)** Quantification of the levels of EXOC1 and EXOC6 in membrane fractions from MCF7 cells and BT549 cells (*n* = 3). **(D)** A heat map showing the abundance of RAB family GTPases in purified membrane fractions from MCF7 cells. **(E)** DHE staining of PHB1-RNAi and PHB1-RNAI; +RAB11-O/E ovarioles. **(F)** quantification of peb antibody staining fluorescence from control and PIPK-RNAi ovarioles (*n* = 20). **(G)** volcano plot depicting the protein level differences between control 3T3cell and 3T3 cells cultured with H_2_O_2_. **(H)** A heat map depicting the expression of NRF2 target genes in cells adjacent to ROS-producing cells. **(I)** a heatmap of the gene expression abundance changes in genes associated with the term “secreted protein.” Expression levels are expressed as a ratio ROS-coculture/control. **(J)** A heatmap of the top 100 most significant differentially regulated genes in cells adjacent to ROS-producing cells. **(K)** A heatmap of the abundance of genes associated with the gene ontology term “extracellular protein” from our list of genes downregulated in cells adjacent to ROS-producing cells(Red text indicates a known role in cancer growth, metastasis, and angiogenesis). **(L)** ROS-Levels in Hek293 control cells, DAAO-expressing, and cells co-cultured adjacent to ROS-producing DAAO+ cells (*n* = 25). **(M)** A Venn diagram showing the overlap of genes known to be regulated by extracellular ROS exposure (NCBI GEO: GSE227554, H_2_O_2_ exposure for 6 h) and our list of genes regulated by co-culture in contact with ROS-producing cells. **(N)** GO term enrichment for the 23 genes overlapping our data and the H_2_O_2_-regulated genes studied in GSE227554. Student’s *t* test was used for all pairwise comparisons, and one-way ANOVA was used for all experiments containing >2 sample groups. Error bars represent the standard deviation.

To determine the mechanism of ROS regulation of protein trafficking, we utilized a membrane proteomics approach to characterize the changes in membrane proteome induced by high ROS levels. Due to limitations in tissue amount and the fact that commonly used *Drosophila* cell lines (S2 and KC cells) do not express the ligand Delta, we utilized mammalian cell lines known to express high levels of Delta-like ligands. Given the highly conserved nature of the Notch signaling pathway, we utilized human and mouse cell models (MCF7 cells and NIH3T3 cells) to determine how ROS exposure impacts membrane-associated proteins involved in protein trafficking. Using human MCF7, which expresses high levels of the Delta-like ligands DLL1 and DLL4 (Wang et al., 2017; Kumar et al., 2019), we examined the relationship between ROS and protein trafficking using membrane proteomics. To examine the impact of ROS on the membrane proteome we transiently treated cells with H_2_O_2_ for 30 min (Fig. 4 A). Membrane proteins were then extracted and subjected to quantitative proteomics. Our analysis measured the relative abundance of ∼3,600 proteins in membrane fractions in these studies. We observed 146 proteins that change abundance at least twofold (FDR < 0.05) in membrane fractions from cells treated with H_2_O_2_ (Fig. 4, B and C; Fig. S5 A; and Table S2). Most of those proteins were downregulated (Fig. 4, B and C). Common membrane proteins, such as SNAP, syntaxin, and VAMP, were unaffected (Fig. 4 D). Interestingly, 131 proteins were depleted from the membranes of cells treated briefly with H_2_O_2_ (Fig. 4, B and C). Among these downregulated proteins, we observed a significant downregulation of all eight core components of the Exocyst complex (EXOC1-8) (Fig. 4 E). The exocyst complex is a crucial regulator of endosomal recycling and the primary mechanism for targeting recycling endosomes to the plasma membrane. The Exocyst complex is also critical in Delta trafficking and Notch activation (Jafar-Nejad et al., 2005; Pushpa et al., 2021; Emery et al., 2005).

We repeated these studies in *Drosophila* ovary tissue using targeted protein LC/MS to assess the abundance of exocyst subunits in membrane fractions. Given that *Phb1* may have multiple roles in the mitochondria, we used ND75-RNAi to induce ROS production by disrupting complex 1 of the ETC. ND75/NDUFS1 has a defined role in ROS production in mammals and *Drosophila*. We examined the effect of ROS production on exocyst membrane association by inducing ROS production (ND75-RNAi) and examining exocyst levels in the membrane using targeted LC/MS mass spec. In this experiment, we were able to detect and measure the levels of four of the eight exocyst components. Interestingly, ROS production causes a similar reduction in all four detectable exocyst complex subunits sec6, sec3, sec15, and exo84 in oocyte membrane extracts (Fig. 4 G). These data support the model that ROS levels, whether through exposure or by production from mitochondrial dysfunction, prevent exocyst membrane binding. These data indicate that ROS plays a conserved role in regulating endosomal recycling.

Using a third system, we confirmed these membrane proteomic experiments in NIH3T3 cells. We observed a similar downregulation of 177 membrane proteins involved with ribosomal function and protein trafficking upon H_2_O_2_ treatment (Fig. 4 F and Table S3). Notably, all seven exocyst subunits detected in our 3T3 cell dataset display a depletion in membrane fractions identical to what we observed in MCF7 cells (Fig. 4 H).

We validated these results by purifying membrane fractions from control, H_2_O_2_ treated, and H_2_O_2_ treated cells that recovered 2 h in media lacking H_2_O_2_. We then measured exocyst subunit levels by using western blots. Consistent with our model, total protein levels for EXOC1, EXOC3, and EXOC6 were unaffected (Fig. 4 J). However, we observed significant reductions in EXOC1, EXOC3, and EXOC6 levels from membrane fractions in H_2_O_2_-treated cells (Fig. 4 J). Exocyst complex subunits did not return to normal during the 2-h recovery period.

When examining known exocyst regulators (Rho, RAL, and CDC42) and other Rab protein family members, we found that they were unaffected in our MCF7 proteomics data set (Fig. 4 I). These data indicate that the exocyst complex’s ability to bind membrane is uniquely sensitive to ROS production by the mitochondria. Intriguingly, we found that specific protein domains were enriched in our downregulated list of membrane proteins (Fig. S5 A). Among those were proteins with armadillo-like folds found in membrane proteins such as clathrin adapters and PI3 kinase. We also found that P-loop-containing nucleoside triphosphate hydrolase-containing proteins, such as Dynamin and ABC (ATP Binding Cassette) transporters, were enriched. Finally, we observed Cullin-repeated-containing domains found in exocyst proteins such as EXOC84 and EXOC70, suggesting that these domains may contribute to the ROS sensitivity of their trafficking.

To test whether cells that endogenously produce more ROS have defects in exocyst membrane binding, we compared a breast cell line that does not produce ROS (MCF7) to a breast cancer line that produces high levels of ROS (BT549) (Fig. S5 B). We found that these cell lines lack Exoc1 and Exoc6 in their membrane fractions (Fig. 4 K and Fig. S5 C) (Exoc3 levels were below the limit of detection in BT549 cells in these experiments). We examined these effects in vivo by comparing exocyst levels in membrane fractions from control liver and Myc-o/e expressing tumors. Myc overexpression is known to induce mitochondrial ROS production in cancer models (Tanaka et al., 2002; Sullivan and Chandel, 2014, Zhou et al., 2014; Biroccio et al., 2001). Consistent with our model, Myc-o/e membrane fractions completely lack Exoc1, Exoc3, and Exoc6 in their membrane fractions (Fig. 4 L). Intriguingly, MYC overexpression does not affect the expression levels of Exocyst complex subunits based on previous transcriptomics studies of this tumor model (Lafita-Navarro et al., 2018).

### Rab11 overexpression rescues *Phb1-RNAi* phenotypes

**Figure 5.**
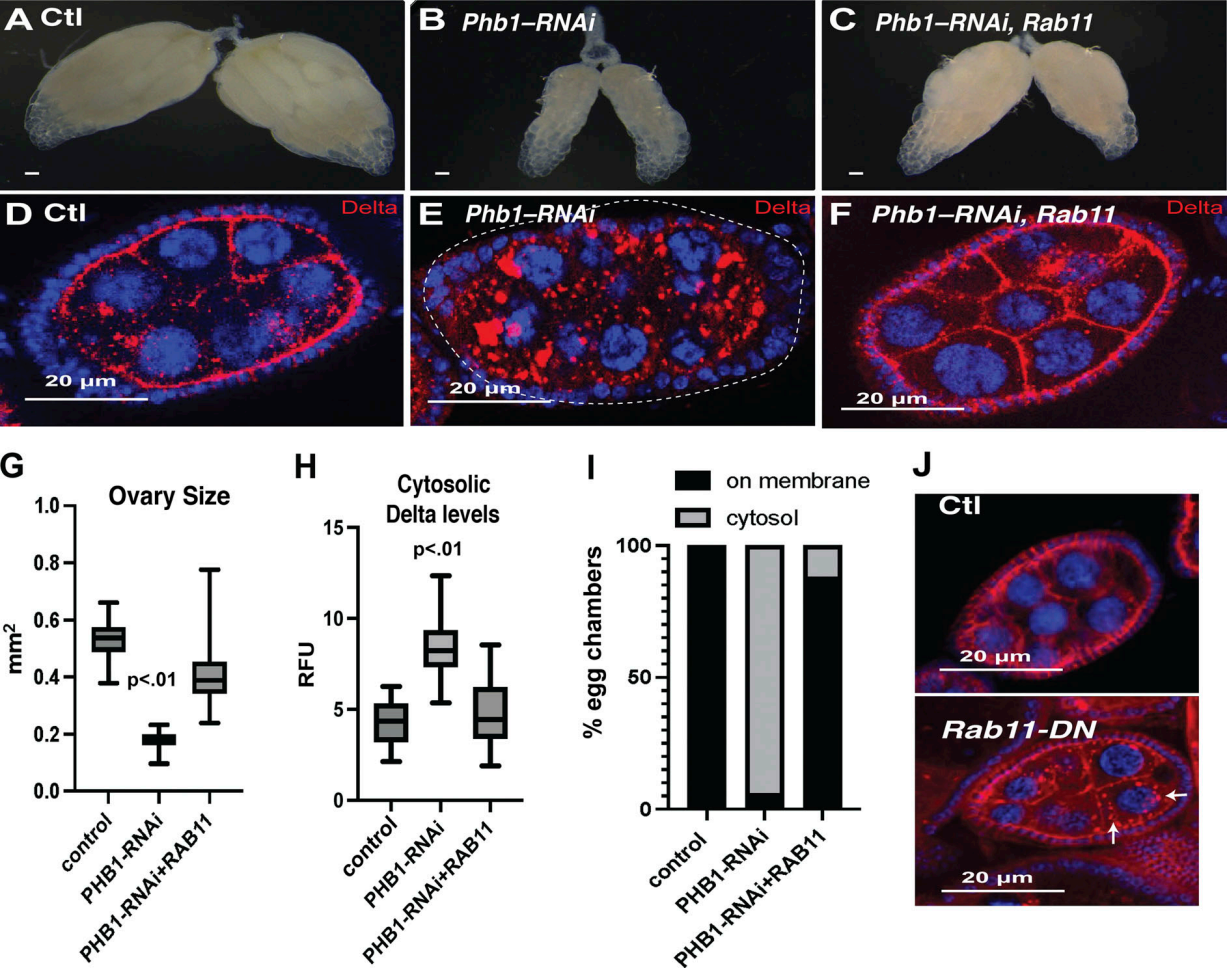
**ROS disrupts exocyst-mediated endosomal recycling of Delta ligands. (A–C)** Whole ovary images of control, PHB1-RNAi, and PHB1-RNAi +RAB11(o/e) egg chambers (two copies of GAL4). **(D–F)** Immunofluorescence images of control, PHB1-RNAi, and PHB1-RNAi +RAB11(o/e) egg chambers stained with Delta antibodies. **(G)** Ovary size measurements of control, PHB1-RNAi, and PHB1-RNAi +RAB11(o/e) egg chambers (one copy of GAL4) (*n* = 20 ovaries). **(H)** cytosolic delta level measurements of control, PHB1-RNAi, and PHB1-RNAi +RAB11(o/e) egg chambers (*n* = 25 egg chambers). **(I)** A graph depicting the percentage of mid-oogenesis egg chambers that display Delta membrane localization or cytosolic Delta aggregates (*N* = 25 egg chamber). **(J)** Immunofluorescence images of control and RAB11-DN expressing egg chambers stained with Delta antibodies (red) Arrows indicate Delta puncta. Student’s *t* test was used for all pairwise comparisons, and one-way ANOVA was used for all experiments containing >2 sample groups. Error bars represent the standard deviation.

These data suggest that defective endosomal recycling causes the developmental phenotypes and Delta trafficking defects seen in *Phb1-RNAi* egg chambers. To test this idea, we overexpressed the key exocyst regulator Rab11 in *Phb1-RNAi* follicles. Rab11 overexpression was sufficient to rescue the small ovary phenotype of *Phb1-RNAi* females (Fig. 5, A–C and G). Rab11-overexpression also rescued the Delta trafficking defects of *Phb1-RNAi* egg chambers, causing reduced cytosolic Delta levels and restoring the Delta on the membrane in over 90% of *Phb1-RNAi* egg chambers (Fig. 5, D–F, H, and I). RAB11 overexpression did not reduce ROS levels (Fig. S5 E), suggesting RAB11 expression bypasses the effects of ROS on endosomal recycling. This rescue is not due to dilution of the GAL4 because UAS-Rab11 did not impact the knockdown efficiency of Phb1 in our RNAi lines (Fig. S1 J). Consistent with previous literature (Emery et al., 2005; Jafar-Nejad et al., 2005), we confirmed that disrupting Rab11-mediated endosomal recycling by expressing Rab11-DN in germ cells causes Delta trafficking defects similar, although weaker, to those we observed in *Phb1-RNAi* egg chambers (Fig. 5 J). This is consistent with mild inhibition of Rab11 by the dominant negative transgene. These data further confirm the role of Rab11/exocyst in Delta trafficking. Moreover, these data support our model that defective exocyst-mediated trafficking is the primary cause of the Delta-Notch signaling defects triggered by mitochondrial ROS production. In addition, these data provide novel insight suggesting that endosomal trafficking is a critical mechanism that drives ROS-associated phenotypes caused by mitochondrial dysfunction.

### ROS reduces cellular PIP2 levels

**Figure 6.**
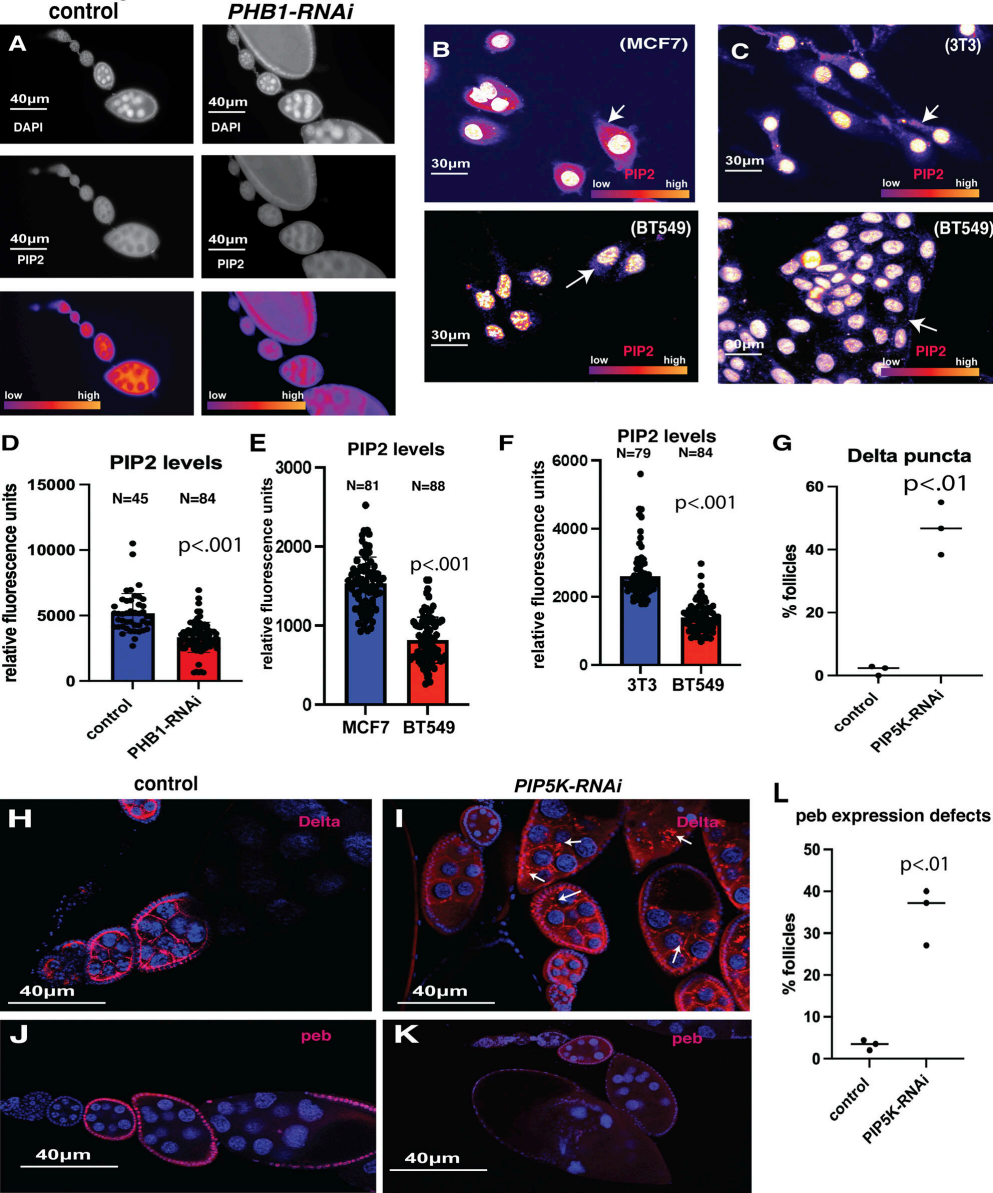
**ROS regulates endosomal recycling by reducing the levels of PIP2 in membranes. (A)** Images of ovarioles from control and PHB-RNAi females. Top image DAPI, middle Images PIP2, bottom image LUT intensity image of PIP2 levels. **(B)** images of (MCF7) and (BT549) cells stained with DAPI and PIP2 antibodies (LUT intensity image of PIP2 levels). **(C)** Images of control (3T3) and (BT549) cells stained with PIP2 antibodies (LUT intensity image of PIP2 levels). Fluorescence levels are expressed with a Fire LUT. Arrows indicate a PIP signal in the cell body. **(D)** Quantification of PIP2 antibody staining levels from control and PHB-RNAi ovarioles. **(E)** Quantification of PIP2 antibody staining fluorescence from (MCF7) and (BT549) cells. **(F)** Quantification of PIP2 antibody staining fluorescence from control (3T3) and (BT549) cells. **(G)** Quantification of the percentage of follicles containing punctate Delta staining from three independent experiments. (Each experiment contained at least 25 egg chambers, and the total number of eggs assayed for each group was at least 85). **(H and I)** Delta antibody staining images from control and PIP5K-RNAi (*sktl*-RNAi) ovarioles. Arrows point to Delta puncta. **(J and K)** peb antibody staining images from control and PIP5K-RNAi (*sktl*-RNAi) ovarioles. **(L)** Quantification of the percentage of follicles containing abnormally low peb expression from three independent experiments. (Each experiment contained at least 25 egg chambers, and the total number of egg chambers assayed for each group was at least 85). Student’s *t* test was used for all pairwise comparisons, and one-way ANOVA was used for all experiments containing >2 sample groups. Error bars represent the standard deviation.

Previous studies have shown that the exocyst complex binds the phospholipid PIP2 to target recycling endosomes to the plasma membrane (Maib and Murray, 2022; Thapa et al., 2012; Synek et al., 2021). Moreover, PIP2 binding domains were enriched in our list of ROS-regulated proteins in purified membrane fractions (Fig. S5 A). Based on these data, we hypothesized that ROS production may influence cellular PIP2 levels to regulate exocyst membrane binding. Using commercially available antibodies for PIP2, we examined PIP2 levels in control and *Phb1-RNAi* egg chambers by immunofluorescence. Compared to controls, *Phb1-RNAi* egg chambers significantly reduce PIP2 levels in germ cells during mid-oogenesis (Fig. 6, A and D). Suggesting PIP2 production in ROS+ *Phb1-RNAi* egg chambers is suppressed. We examined the conservation of this relationship between ROS production and PIP2 levels in mammalian cell lines. Previous studies have shown that triple-negative breast cancer cells (BT549) display very high levels of ROS production when compared to hormone-responsive cells (MCF7) (Sarmiento-Salinas et al., 2019) (Fig. S5 B). Using immunofluorescence, we stained cells with PIP2 antibodies and measured cellular PIP2 levels in BT549 cells and found a significant reduction in PIP2 levels when compared to MCF7 cells and mammalian fibroblast (NIH3T3) cell lines (Fig. 6, B, C, E, and F). Interestingly, PIP2 accumulates at high levels in the nucleus as well as on membranes in mammalian cells, and it is thought to regulate mRNA splicing and chromatin dynamics (Boronenkov et al., 1998; Zhao et al., 1998; Osborne et al., 2001; Gonzales and Anderson, 2006, Mellman et al., 2008). However, both nuclear and membrane PIP2 are reduced in ROS-producing BT549 Cells. To confirm the role of PIP2 in Delta trafficking, we inhibited PIP2 biosynthesis by silencing PIP5K expression via RNAi in *Drosophila* germ cells. Consistent with our model, reducing PIP2 levels in the germ cells partially recapitulates the Delta trafficking defects we observe in PHB1-RNAi oocytes. Where control cells show Delta on the membrane with no puncta in the cytosol, reducing PIP2 levels in germ cells by silencing PIP5K causes Delta puncta to form in the cytosol of roughly 50% of *Phb1-RNAi* egg chambers (Fig. 6, G–I). Furthermore, inhibiting PIP2 biosynthesis in germ cells impaired the expression of the Notch target gene(peb) in adjacent FCs (Fig. 6, J–L and Fig. S5 F). These data are consistent with previous studies that show that ROS production inhibits PIP5K activity in a Syk-dependent manner (Chen et al., 2009). Together, these data support the model that mitochondrial dysfunction impairs cell–cell communication by ROS-mediated suppression of PIP2 levels in the plasma membrane.

### ROS non-cell autonomously suppresses Notch signaling and induces ECM remodeling in adjacent cells

**Figure 7.**
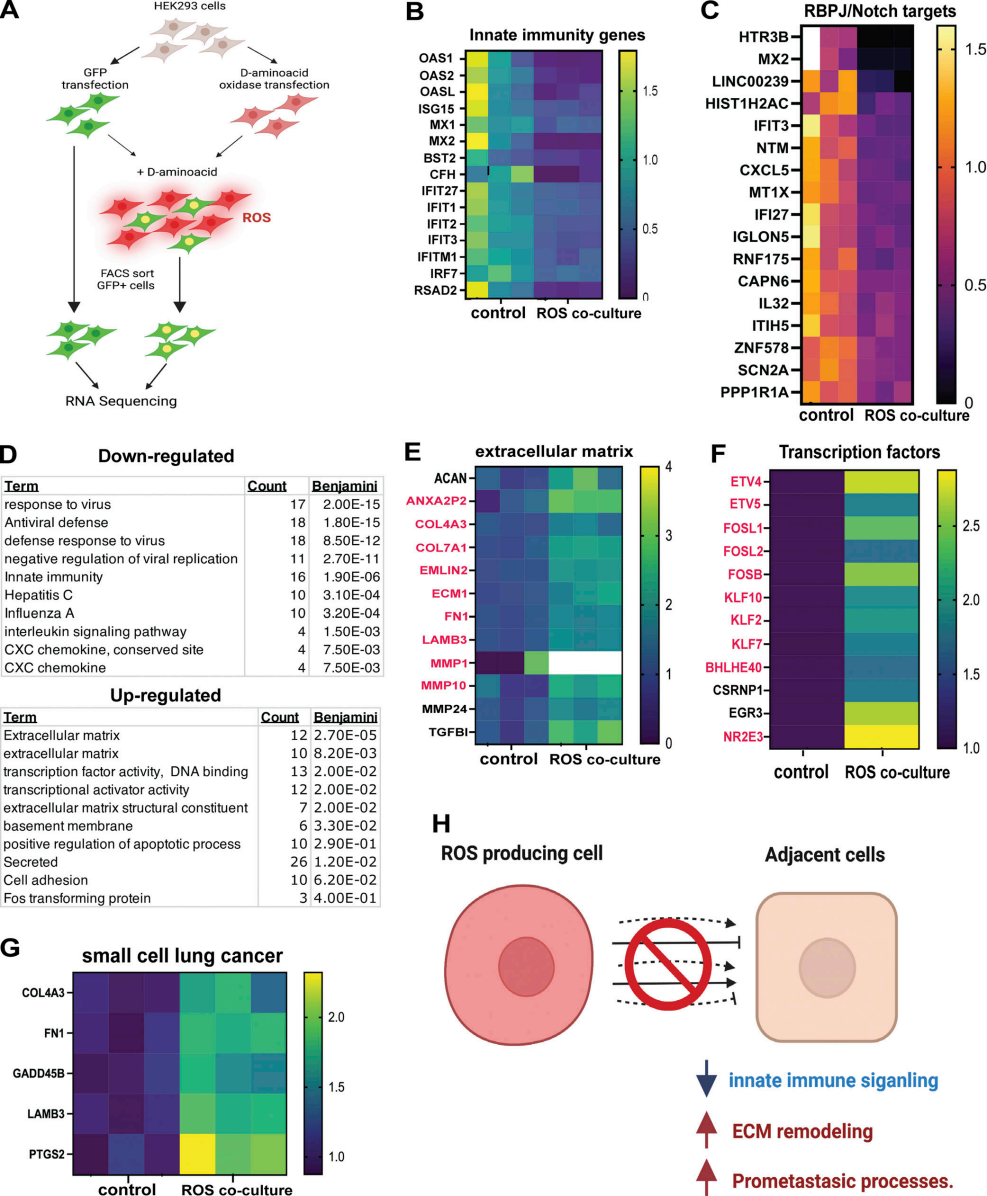
**ROS production non-cell autonomously promotes pro-cancer processes in adjacent cells. (A)** A diagram describing the mammalian cell model used to examine the impact of ROS production on transcription in adjacent cells. In this model, we co-cultured ROS-producing HEK293 cells expressing (DAAO expressing) with HEK293 cells expressing GFP in the presence of D-amino acids for 8 h. We then isolated the GFP+ cells via flow cytometry and purified mRNA for RNA-seq. All subsequent data is based on three independent biological replicates. All genes that were identified as differentially regulated display an FDR < 0.05). **(B)** A heat map depicting the expression ratio (ROS-coculture/control) for genes associated with the GO term innate immunity. **(C)** A heat map depicting the expression ratio (ROS-coculture/control) for genes known to be directly regulated by the RBPL/Notch signaling pathway. **(D)** A table of Gene ontology terms enriched in genes downregulated(top) or upregulated (bottom) by ROS production in adjacent cells. **(E)** A heat map depicting the expression ratio (ROS-coculture/control) for genes associated with the GO term extracellular matrix. (red indicates known association with cancer growth and metastasis). **(F)** A heat map depicting the expression ratio (ROS-coculture/control) for genes associated with the GO term transcription factor (red indicates known association with cancer growth and metastasis). **(G)** A heat map depicting the expression ratio (ROS-coculture/control) for genes associated with the GO term small cell lung cancer. **(H)** A model summarizing the transcriptional changes we observed in cells co-cultured adjacent to ROS-producing cells. Student’s *t* test was used for all pairwise comparisons, and one-way ANOVA was used for all experiments containing >2 sample groups. Error bars represent the standard deviation.

While ROS production can impair the differentiation of adjacent cells in the ovary, it remains unclear what cellular processes are the first to respond to mitochondrial ROS production in neighboring cells. Given the effect on FC differentiation, many of the transcription changes in *Phb1-RNAi* egg chambers likely stem from the developmental abnormalities we observed in FCs. This made identifying the pathways and processes that make up the initial response to ROS production in adjacent cells unclear. Therefore, we moved to a human cell system (HEK293 cells) to characterize the primary response to non-cell autonomous ROS production. HEK293 cells were chosen because they are epithelial and are known to express human orthologs of Delta (DLL1 and DLL4). We made two cell lines, one a HEK293 line that stably expressed GFP and another that expresses D-amino acid oxidase (DAAO) at high levels. DAAO is an enzyme that consumes non-utilizable D-amino acids to produce ROS (Matlashov et al., 2014). We co-cultured GFP-expressing HEK293 cells with HEK293 that produce ROS from a DAAO transgene (30:70 GFP: DAAO ratio at ∼75% confluence). These conditions were chosen to ensure each GFP+ cell was in direct contact with multiple ROS-producing DAAO cells. After overnight incubation (10 h) supplemented with d-alanine, we collected GFP+ cells via FACS sorting and examined the transcriptional profiles of GFP+ and GFP+ cells co-cultured with ROS-producing cells (Fig. 7 A). This system allowed us to look acutely at the immediate transcriptional response in cells adjacent to ROS-producing cells and determine the regulatory networks that respond acutely to ROS.

We found that cells co-cultured with ROS-producing cells displayed a misregulation of 295 genes. These genes displayed an FDR >0.05 and at least a 1.5-fold change in expression. 138 genes were downregulated, and 157 genes were upregulated (Fig. S5 G). Consistent with our model, 17 genes, shown to be bound by the Notch DNA binding cofactor RBPJ_kappa_ in ChIP-sequencing experiments (Lachmann et al., 2010), are significantly downregulated in cells co-cultured with ROS-producing cells (Fig. 7 C). These RBPJ targets include: HTR3B, MX2, LINC00239, HIST1H2AC, IFIT3, NTM, CXCL5, MT1X, IFI27, IGLON5, RNF175, CAPN6, IL32, ITIH5, ZNF578, SCN2A, and PPP1R1A. These data are consistent with disruptions in Notch signaling when ROS is produced in neighboring cells. Using gene ontology analysis, we found several genes involved in the innate immune response, including genes regulated by interferon, were downregulated, suggesting that ROS production suppresses innate immune signaling in adjacent cells (Fig. 7, B and D). We also observed that several genes encoding secreted proteins, including TNF and CXCL proteins, were differentially regulated, suggesting cell–cell signaling is disrupted by ROS production (Fig. S5, I and K). Consistent with this idea, we observed 41 genes associated with the term “signaling protein” in our downregulated data set. These include genes involved in interleukin signaling, cytokine signaling, and developmental signaling. These data are consistent with our model that ROS production disrupts cell–cell signaling between adjacent cells.

When we examined the genes upregulated by ROS production in adjacent cells, we found that 25 were genes that encode extracellular proteins (Fig. 7 D and Fig. S5 K). Interestingly, ROS production in adjacent cells induces many extracellular matrix components and enzymes that control ECM remodeling (Fig. 7 E). In addition, these genes encode several collagen proteins and matrix metalloproteases (MMPs), suggesting that ROS production from the adjacent cells induces the remodeling of ECM. We also observed 12 transcription factors upregulated when cells are co-cultured with ROS-producing cells. Interestingly, 10 of these 12 transcription factors are associated with roles in cancer growth, metastasis, and angiogenesis (Park et al., 2012; Aytes et al., 2013; Yuan et al., 2014; Qi et al., 2015; Zhang et al., 2016; Li et al., 2018; Luo et al., 2018; Sethuraman et al., 2018; di Martino et al., 2019; Du et al., 2020; Meng et al., 2020; Lin et al., 2021, 2022; Wan et al., 2021; Wang et al., 2021) (Fig. 7 F, red text indicates cancer association). Intriguingly, when we examine all three of these gene ontology categories, we find that 17/25 genes encoding extracellular proteins (Fig. S5 E red text indicates a known cancer association) and 9/12 genes encoding ECM proteins have been implicated in cancer metastasis, growth, and angiogenesis (Ifon et al., 2005; Yen et al., 2009; Yoneda et al., 2010; Park et al., 2012; Al-Alem et al., 2013; Aytes et al., 2013; Jiang et al., 2013; Li et al., 2013, 2018; Klupp et al., 2016; Zhang et al., 2016, 2022a, 2022b; Shen et al., 2017; Lo et al., 2018; Luo et al., 2018; Sethuraman et al., 2018; Tao et al., 2018; di Martino et al., 2019; Jiang et al., 2019; Pucci et al., 2019; Wang et al., 2019, 2021; Ban et al., 2020; De Andrade et al., 2020; Du et al., 2020; Meng et al., 2020; Steinhaeuser et al., 2020; Zhu et al., 2020; He et al., 2022; Lee et al., 2021; Lin et al., 2021, 2022; Oh et al., 2021; Ran et al., 2021; Wan et al., 2021; Sun et al., 2022; Widjaja et al., 2022) (Fig. 7 E). Consistent with this strong association, we also found that ROS production in adjacent cells induces five genes implicated specifically in small-cell lung cancer (Fig. 7 G). These data are consistent with the model that mitochondrial dysfunction can disrupt cell–cell signaling and promote cancer-associated processes in adjacent cells (Fig. 7 H).

To exclude the possibility that ROS passes between cells to mediate the changes in gene expression, we specifically examined the expression of known ROS-responsive genes in cells co-cultured with ROS-producing cells. NRF2 is a critical redox-regulated transcription factor activated by ROS (Ma, 2013). However, NRF2 target gene expression is not affected in our data, indicating that the ROS transcriptional response is not activated in these cells (Fig. S5 H). We also compared our gene expression dataset to publicly available RNA-seq data characterizing ROS-responsive gene expression (NCBI GEO #GSE227554) in human cells. From that data set, over 550 genes are regulated by ROS exposure. Of those genes, only 23 genes are differentially regulated in both data sets (Fig. S5 M), and over 90% of the genes in our data set are distinct from a cell-autonomous transcriptional response to ROS. Among those overlapping genes are primarily basic transcriptional machinery (Fig. S5 N). Moreover, when we measured ROS levels in control cells, DAAO-expressing cells, and ROS co-culture cells, we found that ROS levels did not increase substantially in ROS Co-cultured cells. Together, these data indicate that ROS from DAAO cells is not trafficked between cells to induce a cell-autonomous transcriptional response to ROS. Moreover, these data support our model that the transcriptional response in ROS co-cultured cells differs from the cell-autonomous transcriptional response to ROS.

## Discussion

While mitochondrial dysfunction has apparent intrinsic effects on development and disease progression, much less is known about the non-cell autonomous role of mitochondrial dysfunction in development and disease. Using a multi-platform approach, we have found that mitochondrial dysfunction in germ cells blocks the differentiation of adjacent somatic cells. In addition, we have found that mitochondrial dysfunction causes ROS production that, in turn, reduces PIP2 levels in the cell and disrupts endosomal recycling by displacing the exocyst complex from the membrane. Furthermore, ROS production non-cell autonomously suppresses the viral response, notch signaling, and innate immune signaling in adjacent cells. In contrast, ROS production causes the induction of factors involved in ECM remodeling and several cancer-associated processes in neighboring cells. These studies show that mitochondrial dysfunction elicits profound non-cell autonomous effects on adjacent cells. These defects in cell–cell communication disrupt development and may underlie important aspects of mitochondrial disease (Castro and Freeman, 2001; Alfadda and Sallam, 2012; Gorman et al., 2016, Russell et al., 2020; Shimokawa, 2020; Madan et al., 2022; Rossmann et al., 2021).

Many aspects of cellular function are highly responsive to oxidative stress (Martindale and Holbrook, 2002; Schieber and Chandel 2014; Sies et al., 2017; Forman and Zhang, 2021; Ma, 2013; Gerashchenko et al., 2012; Chaudhari et al., 2014). One challenge in understanding the impact of mitochondrial dysfunction is defining the molecular mechanisms disrupted by ROS production. While ROS can damage DNA (and proteins), oxidize lipids, and induce oxidative stress, the precise cause of pathology can be challenging to identify in many cases. Some studies have shown that mitochondrial dysfunction can induce the unfolded protein response (UPR), impair ER translation, and stimulate ERAD (Eletto et al., 2014; Poplawski et al., 2019). In many cases, disease presentation is considered the summation of all these effects of ROS (Castro and Freeman, 2001; Alfadda and Sallam, 2012; Shimokawa, 2020).

Our study provides an example where a single molecular mechanism accounts for the role of ROS in regulating cellular differentiation. Our data has revealed a surprising link between ROS production and membrane receptor trafficking through the recycling endosome, representing a highly conserved initial response to ROS. Using multiple systems, we have found that ROS levels regulate exocyst complex membrane binding by reducing the levels of PIP2 in the cell. We have shown that the exocyst complex is released from the membrane within minutes of exposing cells to ROS. While other studies have described non-cell-autonomous roles for ROS in other models, our work highlights the impact that ROS-induced defects in protein trafficking can have on models of mitochondrial dysfunction. Our work also indicates that restoring receptor trafficking is a viable strategy to improve tissue function in the face of mitochondrial defects.

Moreover, our data show that overexpressing the key exocyst regulator RAB11 (Emery et al., 2005; Jafar-Nejad et al., 2005; Giagtzoglou et al., 2012) is sufficient to rescue the phenotypes caused by ROS production. These data suggest that exocyst-mediated trafficking is a crucial early response mechanism that couples ROS production to changes in cell–cell signaling in diseases caused by mitochondrial dysfunction. In conjunction, these data further support the idea that phenotypes and pathologies caused by ROS production can stem from a single molecular disruption and not systemic damage.

To complement these studies, we exploited the utility of human cells to systematically define the cellular response to ROS production from adjacent cells. Interestingly, we observed a suppression of notch signaling, innate immunity, and the induction of a gene expression profile that supports ECM remodeling. Since innate immunity can impede cancer growth, suppressing these pathways in cells/tissue could promote tumor growth and metastasis (Corrales et al., 2017; Saleiro and Platanias, 2019). Consistent with these findings, we find ECM remodeling factors such as MMPs and other secreted proteases are significantly upregulated in cells co-cultured with ROS-producing cells. Inducing the expression of these genes would promote ECM remodeling and metastasis/cell migration in tumor models. Overall, we observed 28 genes implicated in cancer growth and metastasis upregulated in cells co-cultured with ROS-producing cells. Importantly, we have found that ROS induces a transcriptional response in adjacent cells distinct from the cell-autonomous response ROS.

Our work defines a highly conserved cellular response to mitochondrial ROS production that can regulate cell–cell communication. Unlike previous studies examining ROS regulation of UPR (Eletto et al., 2014; Poplawski et al., 2019; Rossmann et al., 2021), we have found that restoring endosomal recycling by overexpressing RAB11 rescues the developmental defects caused by mitochondrial ROS production. These data suggest that defective endosomal recycling may significantly contribute to the developmental and cellular defects that underlie many pathologies caused by ROS production. Moreover, our data show that multiple models with high levels of ROS production display defects in Exocyst-mediated endosomal recycling, suggesting that this mechanism plays a crucial role in communication between cells in models of development and disease.

## Materials and methods

All reagents and stocks are listed in Table 1.

**Table 1.** Reagents

Reagent or resource	Source	Identifier
Antibodies		
Mouse monoclonal anti-Notch, extracellular domain (ECD) (Drosophila)	Developmental Studies Hybridoma Bank (DSHB)	C458.2H
Mouse monoclonal anti-delta (Drosophila)	DSHB	C594.9B
Mouse monoclonal anti-tyrosine-protein phosphatase 69D (Ptp69D) (Drosophila)	DSHB	3F11
Rabbit polyclonal anti-PHB	Sigma-Aldrich	HPA003280
Rabbit polyclonal anti-EXOC1	Sigma-Aldrich	HPA037706
Rabbit polyclonal anti-EXOC3	Sigma-Aldrich	HPA037880
Rabbit polyclonal anti-EXOC6	Sigma-Aldrich	HPA036285
Mouse monoclonal anti-ATP5A [15H4C4]	Abcam	Ab14748
Rabbit polyclonal anti-phospho-Histone H3 (Ser10)	Cell Signaling Technology	9701S
Goat anti-Rabbit IgG (H+L) highly cross-adsorbed secondary antibody, Alexa Fluor plus 488	Thermo Fisher Scientific	A32731
Goat anti-mouse IgG (H+L) highly cross-adsorbed secondary antibody, Alexa Fluor plus 488	Thermo Fisher Scientific	A32723
Goat anti-Rabbit IgG (H+L) highly cross-adsorbed secondary antibody, Alexa Fluor plus 594	Thermo Fisher Scientific	A32740
Goat anti-mouse IgG (H+L) highly cross-adsorbed secondary antibody, Alexa Fluor plus 594	Thermo Fisher Scientific	A32742
Mouse monoclonal anti-PIP2	Thermo Fisher Scientific	2C11
Bacterial and virus strains		
Biological samples		
Chemicals, peptides, and recombinant proteins		
Dihydroethidium (DHE)	Sigma-Aldrich	37291; CAS: 104821-25-2
Tetramethylrhodamine ethyl ester perchlorate (TMRE)	Sigma-Aldrich	87917; CAS: 115532-52-0
Critical commercial assays		
ATP determination kit	Thermo Fisher Scientific	A22066
Mem-PER plus membrane protein extraction kit	Thermo Fisher Scientific	89842
D-Alanine	Sigma-Aldrich	A7377
Deposited data		
Massive.ucsd.edu		MSV000091074
NCBI-GEO		GSE223669
NCBI-GEO		GSE227554
Experimental models: Cell lines		
Human: MCF7	ATCC	HTB-22
Mouse: 3T3	ATCC	CRL-1658
HEk293	ATCC	CRL-1573
BT549	ATCC	HTB -122
Experimental models: Organisms/strains		
*D. melanogaster*: RNAi of Phb1: y [1] sc[*] v [1]; P{y[+t7.7] v[+t1.8] = TRiP.HMS00702} attP2	Bloomington Drosophila Stock Center	BDSC:32912
*D. melanogaster*: RNAi of Bor: y [1] sc[*] v [1] sev[21]; P{y[+t7.7] v[+t1.8] = TRiP.HMC06013} attP40	Bloomington Drosophila Stock Center	BDSC:65057
*D. melanogaster*: RNAi of Fib: y [1] v [1]; P{y[+t7.7] v[+t1.8] = TRiP.HMJ02126} attP40	Bloomington Drosophila Stock Center	BDSC:42553
*D. melanogaster*: RNAi of Nop5: y [1] sc[*] v [1] sev[21]; P{y[+t7.7] v[+t1.8] = TRiP.GL00413} attP2	Bloomington Drosophila Stock Center	BDSC:35484
*D. melanogaster*: Overexpression of Sod2: P{w[+mC] = UAS-Sod2.Z}1B, y [1] w [*]; Sod2[Delta02]/CyO	Bloomington Drosophila Stock Center	BDSC:27645
*D. melanogaster*: Overexpression of Rab11: w [*]; P{w[+mC] = UAS-Rab11-GFP}2	Bloomington Drosophila Stock Center	BDSC: 8506
*D. melanogaster*: RNAi of Phb2: y [1] v [1]; P{y[+t7.7] v[+t1.8] = TRiP.HMS02001} attP40/CyO	Bloomington Drosophila Stock Center	BDSC: 40835
*D. melanogaster*: RNAi of CCT1: y [1] sc[*] v [1] sev[21]; P{y[+t7.7] v[+t1.8] = TRiP.HMS00639} attP2	Bloomington Drosophila Stock Center	BDSC: 32854
*D. melanogaster*: RNAi of Proteasome β5: y [1] sc[*] v [1] sev[21]; P{y[+t7.7] v[+t1.8] = TRiP.HMS00119} attP2	Bloomington Drosophila Stock Center	BDSC: 34810
*D. melanogaster*: RNAi of Proteasome α7: y [1] sc[*] v [1] sev[21]; P{y[+t7.7] v[+t1.8] = TRiP.HMS00068} attP2	Bloomington Drosophila Stock Center	BDSC: 33660
*D. melanogaster*: RNAi of Hsc70-4: y [1] sc[*] v [1] sev[21]; P{y[+t7.7] v[+t1.8] = TRiP.HMS00152} attP2/TM3, Sb [1]	Bloomington Drosophila Stock Center	BDSC: 34836
*D. melanogaster*: RNAi of ND75: y [1] sc[*] v [1] sev[21]; P{y[+t7.7] v[+t1.8] = TRiP.HMS00853} attP2	Bloomington Drosophila Stock Center	BDSC: 33910
*D. melanogaster*: RNAi of Cype: y (1), v (1); UAS-cype-RNAi	Lab stock	
*D. melanogaster*: RNAi of Tom22: y (1), v (1); UAS-tom22-RNAi (HMC04737)	Lab stock	
*D. melanogaster*: RNAi of ATPSynCf6: y (1), v (1); UAS-ATPSynCf6-RNAi (HMC03238)	Lab stock	
Strains		
D. melanogaster RNAi of skittles/PIP5K: y (1), v (1); UAS-PIP5K-RNAi (GL00072)	Bloomington Drosophila Stock Center	BDSC: 33910
D. melanogaster y(1), v(1); Rab11(S25N)-YFP	Bloomington Drosophila Stock Center	BDSC: 33910
D. melanogaster. w(1,118);Tub-roGFP TM3	Bloomington Drosophila Stock Center	BDSC:68669
D.Melanogaster: RNAi of Phb1: y [1] sc[*] v [1]; P{y[+t7.7] v[+t1.8] = TRiP.HMS00399} attP2		
Recombinant DNA		
Software and algorithms		
Proteome discoverer v2.4 SP1		
Sequest HT		
Fiji		
GraphPad Prism 10		
MetaboAnalyst 6.0		
Sciex SIMCA		
Other		

### Fly growth conditions

All flies were raised on standard molasses food used in our previous publications. Unless otherwise stated, all flies were grown at 25°C and fed fresh yeast paste for at least 48 h before dissection and sample collection.

### Imaging

#### Scopes

All imaging was done on a Zeiss AxioObserver 7(491917-0001-000) with Apotome or a Leica SP5. The Zeiss scope is equipped with 10x (NA = 0.45) and 20X (NA = 0.8), 40X (NA = 1.3) and 63X (NA = 1.4) objectives. The Leica scope is equipped with 10x (NA = 0.3), 20X (NA = 0.75), 40X (NA = 1.25), 63X (NA = 1.2).

#### Imaging temperature

All imaging was done at room temperature.

#### Fluorochromes and probes

This study used the following fluorochromes and probes: Ant-iRabbit-alexa488, Anti-mouse-alexa488, Anti-Rabbit-Alexa594, anti-mouse alexa594, TMRE, DHE, eGFP, and DAPI.

#### Imaging media

Fixed samples were mounted in Fluoroshield and live samples were imaged in culture media (graces media for Drosophila tissue and DMEM for mammalian cells).

#### Camera info and acquisition software

Images were acquired using a Zeiss Axiocam 503 with (ZEN) Zeiss acquisition software.

#### Imaging processing software

All image processing and analysis were done using Fiji software. During processing, only brightness and contrast were adjusted. No other parameters were changed.

### Human-MYC liver cancer mouse model

MYC-driven liver tumor model was generated by crossing mice transgenic mice expressing MYC under the control of a tetracycline-responsive element (TRE-MYC) with LAP-tTA mice, which expresses a transgene containing the tetracycline-controlled trans-activator protein (tTA). Male breeders with two copies of LAP-tTA and a single copy of TRE-MYC were crossed with WT FVB females to generate LAP-tTA/TRE-MYC mice used for experiments. The breeders were maintained on doxycycline water (1 mg/ml). At birth, females with litters were transferred to a fresh cage with regular water to activate MYC overexpression. In this model, animals develop tumors with 100% penetrance between P30–40 and succumb to the disease by day 65 of age. FVB WT males were used as controls for comparison between WT and MYC overexpression. Both the WT males and MYC overexpression males were 52 days old. Mice were euthanized in a CO_2_ chamber, and the livers were dissected and snap-frozen for analysis.

### Immunofluorescence staining

Ovaries were dissected in PBS with 0.1% BSA at room temperature. Dissected ovaries were washed once with PBS, fixed in 4% FA for 20 min at RT, and then rinsed and blocked with wash buffer (0.2% TritonX-100, 0.5% BSA in PBS) for 1 h at RT. Ovaries were then incubated with primary antibodies dissolved in wash buffer overnight at 4°C. The dilution of primary antibodies was as follows: anti-Delta (C594.9B, 1:50), anti-PIP2 (2C11, 1:100), anti-PTP69D (1:250), and anti-peb (1:15). The ovaries were washed with washing buffer three times for 2 h each. Fluorescent-conjugated secondary antibodies were added (1:500) in washing buffer and incubated overnight at 4°C. The ovaries were washed with washing buffer three times for 2 h each. DNA was stained with 0.1 μg/ml DAPI for 20 min at RT. The ovaries were washed in a washing buffer three times for 10 min each and mounted in Fluoroshield. The ovaries were imaged using both a Zeiss laser-scanning confocal microscope and a Zeiss AxioObserver with apotome.

### Cell culture and co-culture conditions

BT549 cells (ATCC# HTB-122) were cultured in RPMI with 10% FBS and 8 µg/ml insulin. The MCF7 (ATCC# HTB-22) and HEK293 (ATCC# CRL-1523) cells were cultured in high glucose DMEM with 10% FBS. Our cell lines were obtained from ATCC. We titrated d-alanine levels from 10 to 100 µM for our co-culture experiment experiments and assayed viability over 16 h. We chose the highest concentration, which yielded healthy cells (25 µM). We then co-cultured the HEK293 (+GFP) cells with HEK293 (+DAAO) in the presence of 25 µM D-alanine for 10 h. The cells were plated at a 70:30 ratio (DAAO: GFP) at high density to ensure the vast majority of GFP+ cells were contacted by multiple DAAO cells. With assistance from the UTSW flow cytometry core, control and co-cultured cells were FASC sorted for GFP+ cells. RNA was purified using TriPure RNA isolation reagent. The UTSW Next Gen sequencing core constructed single-ended mRNA libraries using the Illumina stranded mRNA library kit. The resulting libraries were then sequenced to a depth of roughly 30 million reads using an Illumina next seq 2000. The reads were mapped using STAR (v2.5.3). Differential gene expression analysis was done using EdgeR. Significance gene expression changes were determined by FDR-adjusted P = value.

### DHE staining and ROS quantification

Ovaries were dissected in Grace’s Medium with 10% FBS. Ovarioles were separated manually. Samples were then transferred to another tube containing 40 μm/l Dihydroethidium (DHE), kept in the dark, rotating for 7 min. Samples were washed with Grace’s Medium twice, 5 min each. Images were taken immediately after washing. Quantification of ROS signal was performed using ImageJ (National Institutes of Health). Intensities of ROS were obtained in the region of cytosol. The mean intensity of nurse cell fluorescence is normalized by the follicle cell fluorescence, given that ROS levels in the follicle cells do not change. These fluorescence-based measurements were also supported by isolating drosophila egg chambers, measuring the glutathione redox state (GSH/GSSG ratio), and measuring the levels of methionine sulphoxide (Oxidized methionine) using LC/MS (see LC/MS section for sample prep methods).

### Calculation of delta staining fluorescence and GFP intensity

After staining, stage 6–7 egg chambers were selected to calculate and quantify the intensity of Delta and GFP using Image J software. To calculate Delta staining intensity in the cytosol of germ cells, the germ cell area was selected manually by drawing a small square covering the area between two nurse cell nuclei, excluding the membrane between the germ cell and follicle cell. Average intensity was calculated by dividing the area by the total intensity of this area. To calculate GFP signaling intensity in follicle cells, random regions of follicle cells were selected in main body follicle cells, and average intensity was calculated using ImageJ. Fluorescence between control and RNAi samples was compared between samples.

### Quantification of the number of pH3-positive cells

For quantifying the number of pH3-positive follicle cells, images for each ovariole were taken by z-staking and merged to cover all potential follicle cells. The total number of pH3-positive follicle cells was counted manually for each ovariole. A minimum of 10 ovarioles was counted in each sample. Significance and figures were calculated and produced by Prism.

### Mitochondrial membrane potential assay

Mitochondrial membrane potential was measured by tetramethyl rhodamine ethyl ester (TMRE) staining. Dissected Drosophila ovaries were stained with 5 nM TMRE in PBS for 10 min at RT. Wash three times with Grace’s Media, 2 min each time. Slides were mounted and Imaged immediately after washing. The intensity of TMRE staining was calculated by measuring the pixel intensity in the perinucleus mitochondrial area using Image J. A small piece of the area around the nurse cell nucleus was selected, and mean pixel intensity was calculated and compared between wild-type and Phb1 RNAi samples.

### Seahorse measurements of OCR

Wild-type and Phb1 RNAi ovaries were dissected and incubated for 2 h at RT in graces media to acclimate the egg chamber to graces media. Five stage 8 egg chambers were randomly selected from each sample and put into one well of seahorse cell culture plate. Four Basel OCR measurements were made per sample. Non-mitochondrial respiration was determined by injecting Rotenone (2 µM final concentration) and Antimycin A (2 µM final concentration). The non-mitochondrial respiration was subtracted from the total to calculate the Basel mitochondrial OCR levels. Three wells were assigned as one group. Each sample was measured three times, and each data point represents the average of those three measurements. Each experiment had three biological replicates and was repeated three times.

### ATP measurement assay

ATP measurement was performed using the ATP Determination Kit (A22066; Invitrogen) following the manufacturer’s instructions. Briefly, dissected fly ovaries were homogenized with ice-cold phenol-TE saturated buffer. 500 ml Homogenization was then transferred to a new tube containing 100 μl chloroform and 75 μl de-ionized water. After being shaken for 20 s and centrifuged at 10,000 *g* for 5 min, the upper aqueous phase was used for measurement with a bioluminescence assay. The assay is based on a luciferase reaction, which consumes ATP and produces light. By making the standard curve, ATP in the samples was determined by comparing them to the ATP standard solution.

### LC/MS metabolomics

Samples of 300 oocytes were washed 3x with fresh 1xPBS. Samples were then flash-frozen in liquid nitrogen and stored at −80°C until analysis. Samples were weighed and then homogenized in 1 ml of methanol: H_2_O (80:20). Samples were vortexed for 2 min and then centrifuged ∼20,000 *g* for 15 min. The supernatant was dried down by a low temp speed vac. The resulting dried samples were then frozen at −80°C until analyzed. Samples are analyzed by targeted LC/MS metabolomics to quantify the levels of GSSG, GSH, and Methionine sulphoxide with the assistance of the UT Southwestern metabolomics facility. For Q-TOF mass spectrometer analysis, we reconstituted metabolite pellets in 0.1% formic acid in water and vortexed for 1 min. We then spun the samples at 20,160 *g* at 4°C for 15 min. The resulting supernatants were then loaded into auto-sampler vials for analysis. Data acquisition was performed by reverse-phase chromatography on a 1290 UHPLC liquid chromatography (LC) system interfaced with a 6,550 iFunnel Q-TOF mass spectrometer (MS) (Agilent Technologies). The MS was operated in both positive and negative (ESI+ and ESI−) modes. COmpounds are separated on an Acquity UPLC HSS T3 column. The composition of mobile phase 1 was 0.1% formic acid in water and mobile phase B composition was 0.1% formic acid in 100% ACN. The LC gradient was 0 min: 1% B; 5 min: 5% B; 15 min: 99%; 23 min: 99%; 24 min: 1%; 25 min: 1%. The flow rate was 250 μl min^−1^. The 5 μl of the sample is injected. We utlized the following ESI unit setting: gas temperature 225°C and flow 18 L min^−1^, fragmentor voltage 175 V, sheath gas temperature 350°C and flow 12 L min^−1^, nozzle voltage 500 V, and capillary voltage +3,500 V in positive mode and −3,500 V in negative. The instrument was set to acquire over the full m/z range of 40–1,700 in both modes, with the MS acquisition rate of 1 spectrum s^−1^ in profile format. We used Profinder B.08.00 SP3 software (Agilent Technologies) to process our raw sample data. We have an in-house database containing retention time and accurate mass information on 600 standards from Mass Spectrometry Metabolite Library (IROA Technologies) to identify peaks in our data. Once processed by the core facility, we analyze the data using the following pipeline. Using Sciex SIMCA software, we analyze these data sets and perform Partial Least Square analysis to examine sample clustering. From the PLS analysis, we identify metabolites that exhibit a high VIP score (>1.0) as candidate compounds that contribute to the differential clustering. Subsequent data analysis is done using MetaboAnalyst software 6.0. The data is compiled from eight independent samples/conditions to accurately represent the biological variation of the experiment and meet standards in the field.

### Quantification of the number of follicle cells at stage 10

Ovaries were stained with DAPI. Multiple pictures were taken in a Z-stack to capture all the follicle cells at one side of stage 10 egg chambers. Stacks were Z-projected and used to count DAPI-positive follicle cell nuclei from the merged picture manually. All follicle cells at stage 10 were calculated by doubling the number counted from one side of the egg chamber.

### Measurement of the stage 14 egg size and follicle cell nucleus size

Stage 14 egg size and follicle cell nucleus size were measured using ImageJ software. To exclude the dorsal appendage, the outline of each stage 14 egg chamber was selected manually, the area was measured, and the units of measurement were changed from pixels to square millimeters. To calculate the size of the follicle cell nucleus, the area of the nucleus from stage 14 follicle cells was automatically selected by thresholding and measured using image J. The average size of all follicle cells from one stage 14 egg chamber was calculated. At least eight average nuclei were calculated for each sample.

### Measurement of the size of the ovary

Whole ovaries from wild type, Phb1 RNAi, and rescued lines were captured at bright field light microscopes. Single ovaries from these pictures were manually selected by Image J. The selected area was set as Area of Interest (ROI) and measured by Image J. The Pixel ROI size was then transformed into square millimeters to calculate the exact size of each ovary.

### Membrane protein identification by mass spectrometry

#### Drosophila membrane sample collection

Two hundred pairs of adult ovaries were dissected in graces media. Non-ovary tissue was removed, and samples were rinsed in cold graces media three times. The resulting samples were processed, and membrane proteins were extracted using the Membrane Protein Extraction Kit (Thermo Fisher Scientific) as described below.

Membrane proteins were extracted with a Membrane Protein Extraction Kit (89842; Thermo Fisher Scientific) following the manufacturer’s instructions. MCF7 or 3T3 cells were treated with 1 mM/l H_2_O_2_ for 1 h. The membrane proteins were extracted from 1 × 10^7^ control and treated cells. Protein concentrations were measured, and concentrations were set to 1 µg/µl. 10% (10 µg) of all the extracted proteins were loaded onto the gel and run 1 cm into the gel. The resulting 10 mm gel slice was cut and sent for Mass Spectrometry identification and quantification. Samples were digested overnight with trypsin (Pierce) following reduction and alkylation with DTT and iodoacetamide (Sigma-Aldrich). The samples then underwent solid-phase extraction cleanup with an Oasis HLB plate (Waters), and the resulting samples were injected into an Orbitrap Fusion Lumos mass spectrometer coupled to an Ultimate 3000 RSLC-Nano liquid chromatography system. Samples were injected onto a 75 µm i.d., 75-cm long EasySpray column (Thermo Fisher Scientific) and eluted with a gradient from 0 to 28% buffer B over 90 min. Buffer A contained 2% (vol/vol) ACN and 0.1% formic acid in water, and buffer B contained 80% (vol/vol) ACN, 10% (vol/vol) trifluoroethanol, and 0.1% formic acid in water. The mass spectrometer operated in positive ion mode with a source voltage of 2.0 kV and an ion transfer tube temperature of 275°C. MS scans were acquired at 120,000 resolutions in the Orbitrap, and up to 10 MS/MS spectra were obtained in the ion trap for each full spectrum acquired using higher-energy collisional dissociation (HCD) for ions with charges 2–7. Dynamic exclusion was set for 25 s after an ion was selected for fragmentation.

Raw MS data files were analyzed using Proteome Discoverer v2.4 SP1 (Thermo Fisher Scientific), with peptide identification performed using Sequest HT searching against the human protein database from UniProt. Fragment and precursor tolerances of 10 ppm and 0.6 Da were specified, and three missed cleavages were allowed. Carbamidomethylation of Cys was set as a fixed modification, with oxidation of Met set as a variable modification. The false-discovery rate (FDR) cutoff was 1% for all peptides.

### Analysis of proteomic data

Protein was quantified using a label-free method, which measured the relative intensity of identified peptides. The quantified proteins were then normalized to each sample’s total protein and total ion count. A Volcano diagram and clustered heatmap were drawn using Python. Twofold protein changes with an FDR P value <0.01 were considered significant and selected for further validation. Total cytosol and membrane cellular fractionations were extracted from MCF7 to validate the proteomic data. Cellular lysate with different fractionations was loaded on SDS-PAGE, and a western blot was performed with exoc1, exoc3, and exoc6 antibodies.

### RNA sequencing analysis

RNA samples were isolated using a Tripure RNA isolation reagent (Thermo Fisher Scientific). RNA levels and quality were assayed by Qubit Fluorometer and Agilent Bioanalyzer, respectively. RNA-seq libraries were generated using the TrueSeq Stranded mRNA Sample Preparation Kit. RNA-seq libraries were sequenced using Illumina NextSeq, which generated at least 25 million reads per sample. FastQ files were quality checked using fastqc, and reads were mapped using STAR (v2.5.3a). Read counts were generated using featureCounts, and the differential expression analysis was performed using edgeR. All RNA libraries were collected from independent biological replicates. A at FDR <0.05 and a twofold cut-off were used to identify significantly differentially regulated genes. Z-scores were used to compare data across samples.

### FACS

FACS sorting for CMV-GFP+ HEK293 cells was conducted by the UTSW Immunology flow cytometry core facility. Cells were trypsinized and collected. The cells were then rinsed three times in 1XPBS and resuspended in fresh media (DMEM + 5% FBS). GFP+ Cells were then sorted on a Cellstream flow cytometer. GFP signal was collected in the FL1 channel. Post-sorting RNA was isolated from sorted cells using a Tripure RNA isolation reagent.

### Q-PCR

Ovaries were dissected (20 pairs) and stage 14 egg chamber were collected. The follicle cells were removed using mild bleaching. RNA was then extracted using a Tripure RNA isolation reagent. We then used iTAQ universal SYBR green super mix to measure Phb1 mRNA levels. ACT5C levels were used for normalization. Using the following primers:

Phb1 = Fp 5′-GCA​TCA​AGG​AGA​ACG​TGG​TC-3′ and Rp 5′-TAG​ATC​TTG​GGC​AGC​TGG​TC-3′, Phb2 = Fp 5′-TGT​TCA​GGC​TGA​GGG​AGA​AG-3′ and Rp 5′-CCG​CCG​ACA​AAT​AGA​CCT​TG-3′.

MTPalpha = Fp 5′-CCT​CGA​ACG​GTC​TCT​ATC​CC-3′ and Rp 5′-ACG​GAA​CAG​GGC​AAT​CAA​AC-3′.

Wal = Fp 5′-GGC​CTG​AAG​TCC​GGA​GAT​AA-3′ and Rp 5′-GGA​TCC​TTG​TTG​ATG​GCC​AC-3′.

ND75 = Fp 5′-GAG​AAG​AGT​CCC​AAG​CCA​GT and Rp 5′-GCC​CGT​GTA​GTT​AAT​GTC​CG-3′.

PIP5k = Fp 5′-GTA​GCC​GCT​CTT​CAT​TTG​GG-3′ and Rp 5′-GTG​GGC​TTG​AGT​AGG​TGA​GT-3′.

The samples were analyzed using a Bio-Rad CFX OPUS q-PCR machine.

### DAAO Drosophila transgenic line

The Rhodosporiidum DAAO cDNA was synthesized with CACC added to the 5′end of the insert (GenBank accession# U60066). The cDNA was then cloned into the pENTR d-topo entry using a Thermo D-TOPO cloning kit (# K240020; Thermo Fisher Scientific). The cDNA was then transferred to the pPW (UASp) destination vector by LR-Clonase reaction. The resulting vector was sequenced, purified, and sent off for injection. The transgenic lines were made and mapped to chromosomes by BestGene Inc. DAAO was expressed under the control of mata-GAL4. To induce ROS production flies were fed food supplemented with 1 mg/ml D-alanine to provide substrate for the DAAO reaction.

### Statistics

Significance values in the figures were calculated using either the Student’s *t* test or one-way ANOVA using GraphPad Prism. All experiments were repeated at least three times on independent sets of biological samples. Individual *n* values are listed in the figure legends.

### Data exclusion

All data where both positive and negative controls worked and appeared normal were used in this study. No data were excluded.

### Online supplemental material

Fig. S1 provides an additional characterization of the developmental phenotypes associated with Germline ROS production. In Fig. S2, we provide additional controls and support evidence for the Delta trafficking defects we observed in Phb-RNAi egg chambers. Fig. S3 provides additional data supporting that mitochondrial defects, not developmental arrest, cause defects in Delta trafficking. We provide further evidence for the relationship between ROS production and Delta trafficking in Fig. S4. Fig. S5 offers additional analysis and supporting evidence for our proteomics studies and mammalian cell RNA-seq data. Table S1 is a master list of the gene expression changes observed in Phb-RNAi egg chambers. Table S2 is a master list of the proteins that significantly change their levels in membrane fractions of MCF7 cells treated with ROS. Table S3 is a master list of the proteins that significantly change their levels in membrane fractions of NIN3T3 cells treated with ROS.

## Supplementary Material

Table S1 is an Excel file of the RNA-sequencing data comparing gene expression in control and Phb1-RNAi (one copy) egg chambers.

Table S2 is an Excel file of the proteomics data examining membrane fractions from MCF7 cells treated with H_2_O_2_ for 30 min.

Table S3 is an Excel file of the proteomics data examining membrane fractions from NIH3T3 cells treated with H_2_O_2_ for 30 min.

SourceData F4 is the source file for Fig. 4.

## Data Availability

Our LC/MS proteomics data sets are available at MassIVE(UCSD) under dataset number # MSV000091074. Our RNA-seq data has been deposited at the NCBI GEO under accession number #GSE223669. All data will be placed in source data and provided when requested.

## References

[bib1] Al-Alem, L.F., L.A. McCord, R.C. Southard, M.W. Kilgore, and T.E. Curry Jr. 2013. Activation of the PKC pathway stimulates ovarian cancer cell proliferation, migration, and expression of MMP7 and MMP10. Biol. Reprod. 89:73. 10.1095/biolreprod.112.102327 23843242 PMC4094197

[bib2] Alfadda, A.A., and R.M. Sallam. 2012. Reactive oxygen species in health and disease. J. Biomed. Biotechnol. 2012:936486. 10.1155/2012/936486 22927725 PMC3424049

[bib3] Assa-Kunik, E., I.L. Torres, E.D. Schejter, D.S. Johnston, and B.Z. Shilo. 2007. Drosophila follicle cells are patterned by multiple levels of Notch signaling and antagonism between the Notch and JAK/STAT pathways. Development. 134:1161–1169. 10.1242/dev.02800 17332535

[bib4] Aytes, A., A. Mitrofanova, C.W. Kinkade, C. Lefebvre, M. Lei, V. Phelan, H.C. LeKaye, J.A. Koutcher, R.D. Cardiff, A. Califano, . 2013. ETV4 promotes metastasis in response to activation of PI3-kinase and Ras signaling in a mouse model of advanced prostate cancer. Proc. Natl. Acad. Sci. USA. 110:E3506–E3515. 10.1073/pnas.1303558110 23918374 PMC3773788

[bib5] Ban, Y., P. Tan, J. Cai, J. Li, M. Hu, Y. Zhou, Y. Mei, Y. Tan, X. Li, Z. Zeng, . 2020. LNCAROD is stabilized by m6A methylation and promotes cancer progression via forming a ternary complex with HSPA1A and YBX1 in head and neck squamous cell carcinoma. Mol. Oncol. 14:1282–1296. 10.1002/1878-0261.12676 32216017 PMC7266281

[bib6] Berg, C.A. 2005. The Drosophila shell game: Patterning genes and morphological change. Trends Genet. 21:346–355. 10.1016/j.tig.2005.04.010 15922834

[bib7] Biroccio, A., B. Benassi, S. Amodei, C. Gabellini, D. Del Bufalo, and G. Zupi. 2001. c-Myc down-regulation increases susceptibility to cisplatin through reactive oxygen species-mediated apoptosis in M14 human melanoma cells. Mol. Pharmacol. 60:174–182. 10.1124/mol.60.1.174 11408612

[bib8] Boronenkov, I.V., J.C. Loijens, M. Umeda, and R.A. Anderson. 1998. Phosphoinositide signaling pathways in nuclei are associated with nuclear speckles containing pre-mRNA processing factors. Mol. Biol. Cell. 9:3547–3560. 10.1091/mbc.9.12.3547 9843587 PMC25675

[bib9] Cao, X., and Y. Chen. 2009. Mitochondria and calcium signaling in embryonic development. Semin. Cell Dev. Biol. 20:337–345. 10.1016/j.semcdb.2008.12.014 19530305

[bib10] Castro, L., and B.A. Freeman. 2001. Reactive oxygen species in human health and disease. Nutrition. 17:161, 163–165. 10.1016/S0899-9007(00)00570-0 11240347

[bib11] Chaudhari, N., P. Talwar, A. Parimisetty, C. Lefebvre d’Hellencourt, and P. Ravanan. 2014. A molecular web: Endoplasmic reticulum stress, inflammation, and oxidative stress. Front. Cell. Neurosci. 8:213. 10.3389/fncel.2014.00213 25120434 PMC4114208

[bib12] Chen, M.Z., X. Zhu, H.Q. Sun, Y.S. Mao, Y. Wei, M. Yamamoto, and H.L. Yin. 2009. Oxidative stress decreases phosphatidylinositol 4,5-bisphosphate levels by deactivating phosphatidylinositol- 4-phosphate 5-kinase beta in a Syk-dependent manner. J. Biol. Chem. 284:23743–23753. 10.1074/jbc.M109.036509 19553680 PMC2749148

[bib13] Chen, T., D. Li, Y. Wang, X. Shen, A. Dong, C. Dong, K. Duan, J. Ren, W. Li, G. Shu, . 2023. Loss of NDUFS1 promotes gastric cancer progression by activating the mitochondrial ROS-HIF1α-FBLN5 signaling pathway. Br. J. Cancer. 129:1261–1273. 10.1038/s41416-023-02409-5 37644092 PMC10575981

[bib14] Corrales, L., V. Matson, B. Flood, S. Spranger, and T.F. Gajewski. 2017. Innate immune signaling and regulation in cancer immunotherapy. Cell Res. 27:96–108. 10.1038/cr.2016.149 27981969 PMC5223230

[bib15] Coumailleau, F., M. Fürthauer, J.A. Knoblich, and M. González-Gaitán. 2009. Directional Delta and Notch trafficking in Sara endosomes during asymmetric cell division. Nature. 458:1051–1055. 10.1038/nature07854 19295516

[bib16] De Andrade, W.P., L. Da Conceicao Braga, N.G. Goncales, L.M. Silva, and A.L. Da Silva Filho. 2020. HSPA1A, HSPA1L and TRAP1 heat shock genes may be associated with prognosis in ovarian epithelial cancer. Oncol. Lett. 19:359–367. 10.3892/ol.2019.11095 31897148 PMC6923843

[bib17] di Martino, E., O. Alder, C.D. Hurst, and M.A. Knowles. 2019. ETV5 links the FGFR3 and Hippo signalling pathways in bladder cancer. Sci. Rep. 9:5740. 10.1038/s41598-018-36456-3 30952872 PMC6450944

[bib18] Du, J., L. Fu, F. Ji, C. Wang, S. Liu, and X. Qiu. 2020. FosB recruits KAT5 to potentiate the growth and metastasis of papillary thyroid cancer in a DPP4-dependent manner. Life Sci. 259:118374. 10.1016/j.lfs.2020.118374 32891613

[bib19] Eletto, D., E. Chevet, Y. Argon, and C. Appenzeller-Herzog. 2014. Redox controls UPR to control redox. J. Cell Sci. 127:3649–3658. 10.1242/jcs.153643 25107370

[bib20] Emery, G., A. Hutterer, D. Berdnik, B. Mayer, F. Wirtz-Peitz, M.G. Gaitan, and J.A. Knoblich. 2005. Asymmetric Rab 11 endosomes regulate delta recycling and specify cell fate in the Drosophila nervous system. Cell. 122:763–773. 10.1016/j.cell.2005.08.017 16137758

[bib21] Forman, H.J., and H. Zhang. 2021. Targeting oxidative stress in disease: Promise and limitations of antioxidant therapy. Nat. Rev. Drug Discov. 20:689–709. 10.1038/s41573-021-00233-1 34194012 PMC8243062

[bib22] Gerashchenko, M.V., A.V. Lobanov, and V.N. Gladyshev. 2012. Genome-wide ribosome profiling reveals complex translational regulation in response to oxidative stress. Proc. Natl. Acad. Sci. USA. 109:17394–17399. 10.1073/pnas.1120799109 23045643 PMC3491468

[bib23] Giagtzoglou, N., S. Yamamoto, D. Zitserman, H.K. Graves, K.L. Schulze, H. Wang, H. Klein, F. Roegiers, and H.J. Bellen. 2012. dEHBP1 controls exocytosis and recycling of Delta during asymmetric divisions. J. Cell Biol. 196:65–83. 10.1083/jcb.201106088 22213802 PMC3255984

[bib24] Gonzales, M.L., and R.A. Anderson. 2006. Nuclear phosphoinositide kinases and inositol phospholipids. J. Cell. Biochem. 97:252–260. 10.1002/jcb.20655 16267839

[bib25] Gorman, G.S., P.F. Chinnery, S. DiMauro, M. Hirano, Y. Koga, R. McFarland, A. Suomalainen, D.R. Thorburn, M. Zeviani, and D.M. Turnbull. 2016. Mitochondrial diseases. Nat. Rev. Dis. Primers. 2:16080. 10.1038/nrdp.2016.80 27775730

[bib26] Haynes, P.R., E.S. Pyfrom, Y. Li, C. Stein, V.A. Cuddapah, J.A. Jacobs, Z. Yue, and A. Sehgal. 2024. A neuron-glia lipid metabolic cycle couples daily sleep to mitochondrial homeostasis. Nat. Neurosci. 27:666–678. 10.1038/s41593-023-01568-1 38360946 PMC11001586

[bib27] He, S., Y. Feng, W. Zou, J. Wang, G. Li, W. Xiong, Y. Xie, J.A. Ma, and X. Liu. 2022. The role of the SOX9/lncRNA ANXA2P2/miR-361-3p/SOX9 regulatory loop in cervical cancer cell growth and resistance to cisplatin. Front. Oncol. 11:784525. 10.3389/fonc.2021.784525 35083143 PMC8784813

[bib28] Hirt, H. 2016. Aquaporins link ROS Signaling to plant immunity. Plant Physiol. 171:1540. 10.1104/pp.16.00433 27385821 PMC4936576

[bib29] Hudson, A.M., and L. Cooley. 2014. Methods for studying oogenesis. Methods. 68:207–217. 10.1016/j.ymeth.2014.01.005 24440745 PMC4048766

[bib30] Ifon, E.T., A.L. Pang, W. Johnson, K. Cashman, S. Zimmerman, S. Muralidhar, W.Y. Chan, J. Casey, and L.J. Rosenthal. 2005. U94 alters FN1 and ANGPTL4 gene expression and inhibits tumorigenesis of prostate cancer cell line PC3. Cancer Cell Int. 5:19. 10.1186/1475-2867-5-19 15972109 PMC1200560

[bib31] Ioannou, M.S., J. Jackson, S.H. Sheu, C.L. Chang, A.V. Weigel, H. Liu, H.A. Pasolli, C.S. Xu, S. Pang, D. Matthies, . 2019. Neuron-Astrocyte metabolic coupling protects against activity-induced fatty acid toxicity. Cell. 177:1522–1535.e14. 10.1016/j.cell.2019.04.001 31130380

[bib32] Jafar-Nejad, H., H.K. Andrews, M. Acar, V. Bayat, F. Wirtz-Peitz, S.Q. Mehta, J.A. Knoblich, and H.J. Bellen. 2005. Sec15, a component of the exocyst, promotes notch signaling during the asymmetric division of Drosophila sensory organ precursors. Dev. Cell. 9:351–363. 10.1016/j.devcel.2005.06.010 16137928

[bib33] Jiang, C.P., B.H. Wu, S.P. Chen, M.Y. Fu, M. Yang, F. Liu, and B.Q. Wang. 2013. High COL4A3 expression correlates with poor prognosis after cisplatin plus gemcitabine chemotherapy in non-small cell lung cancer. Tumour Biol. 34:415–420. 10.1007/s13277-012-0565-2 23108892

[bib34] Jiang, K., H. Liu, D. Xie, and Q. Xiao. 2019. Differentially expressed genes ASPN, COL1A1, FN1, VCAN and MUC5AC are potential prognostic biomarkers for gastric cancer. Oncol. Lett. 17:3191–3202. 10.3892/ol.2019.9952 30867749 PMC6396260

[bib35] Klupp, F., L. Neumann, C. Kahlert, J. Diers, N. Halama, C. Franz, T. Schmidt, M. Koch, J. Weitz, M. Schneider, and A. Ulrich. 2016. Serum MMP7, MMP10 and MMP12 level as negative prognostic markers in colon cancer patients. BMC Cancer. 16:494. 10.1186/s12885-016-2515-7 27431388 PMC4950722

[bib36] Kopan, R. 2012. Notch signaling. Cold Spring Harb. Perspect. Biol. 4:a011213. 10.1101/cshperspect.a011213 23028119 PMC3475170

[bib37] Kumar, S., R.K. Srivastav, D.W. Wilkes, T. Ross, S. Kim, J. Kowalski, S. Chatla, Q. Zhang, A. Nayak, M. Guha, . 2019. Estrogen-dependent DLL1-mediated Notch signaling promotes luminal breast cancer. Oncogene. 38:2092–2107. 10.1038/s41388-018-0562-z 30442981 PMC6756232

[bib38] Lachmann, A., H. Xu, J. Krishnan, S.I. Berger, A.R. Mazloom, and A. Ma’ayan. 2010. ChEA: Transcription factor regulation inferred from integrating genome-wide ChIP-X experiments. Bioinformatics. 26:2438–2444. 10.1093/bioinformatics/btq466 20709693 PMC2944209

[bib39] Lafita-Navarro, M.C., M. Kim, N. Borenstein-Auerbach, N. Venkateswaran, Y.H. Hao, R. Ray, T. Brabletz, P.P. Scaglioni, J.W. Shay, and M. Conacci-Sorrell. 2018. The aryl hydrocarbon receptor regulates nucleolar activity and protein synthesis in MYC-expressing cells. Genes Dev. 32:1303–1308. 10.1101/gad.313007.118 30254109 PMC6169836

[bib40] Lee, H., W.J. Kim, H.G. Kang, J.H. Jang, I.J. Choi, K.H. Chun, and S.J. Kim. 2021. Upregulation of LAMB1 via ERK/c-Jun Axis promotes gastric cancer growth and motility. Int. J. Mol. Sci. 22:626. 10.3390/ijms22020626 33435161 PMC7826975

[bib41] Li, D., P. Wei, Z. Peng, C. Huang, H. Tang, Z. Jia, J. Cui, X. Le, S. Huang, and K. Xie. 2013. The critical role of dysregulated FOXM1-PLAUR signaling in human colon cancer progression and metastasis. Clin. Cancer Res. 19:62–72. 10.1158/1078-0432.CCR-12-1588 23136192 PMC3537853

[bib42] Li, H., J. Janssens, M. De Waegeneer, S.S. Kolluru, K. Davie, V. Gardeux, W. Saelens, F.P.A. David, M. Brbic, K. Spanier, . 2022. Fly cell atlas: A single-nucleus transcriptomic atlas of the adult fruit fly. Science 375:eabk2432. 10.1126/science.abk2432 35239393 PMC8944923

[bib43] Li, S., X.D. Fang, X.Y. Wang, and B.Y. Fei. 2018. Fos-like antigen 2 (FOSL2) promotes metastasis in colon cancer. Exp. Cell Res. 373:57–61. 10.1016/j.yexcr.2018.08.016 30114390

[bib44] Lima, T.R.R., E. de Oliveira Lima, J. Delafiori, R. Ramos Catharino, J.L. Viana de Camargo, and L.C. Pereira. 2022. Molecular signatures associated with diuron exposure on rat urothelial mitochondria. Toxicol. Mech. Methods. 32:628–635. 10.1080/15376516.2022.2062271 35379061

[bib45] Lin, J., S. Zhai, S. Zou, Z. Xu, J. Zhang, L. Jiang, X. Deng, H. Chen, C. Peng, J. Zhang, and B. Shen. 2021. Positive feedback between lncRNA FLVCR1-AS1 and KLF10 may inhibit pancreatic cancer progression via the PTEN/AKT pathway. J. Exp. Clin. Cancer Res. 40:316. 10.1186/s13046-021-02097-0 34635142 PMC8507233

[bib46] Lin, Y.M., K.T. Yeh, C.M. Yeh, M.S. Soon, and L.S. Hsu. 2022. KLF10 functions as an independent prognosis factor for gastric cancer. Medicina (Kaunas). 58:711. 10.3390/medicina58060711 35743973 PMC9228861

[bib47] Liu, L., K. Zhang, H. Sandoval, S. Yamamoto, M. Jaiswal, E. Sanz, Z. Li, J. Hui, B.H. Graham, A. Quintana, and H.J. Bellen. 2015. Glial lipid droplets and ROS induced by mitochondrial defects promote neurodegeneration. Cell. 160:177–190. 10.1016/j.cell.2014.12.019 25594180 PMC4377295

[bib48] Lo, P.K., Y. Yao, J.S. Lee, Y. Zhang, W. Huang, M.A. Kane, and Q. Zhou. 2018. LIPG signaling promotes tumor initiation and metastasis of human basal-like triple-negative breast cancer. Elife. 7:e31334. 10.7554/eLife.31334 29350614 PMC5809145

[bib49] Lopez-Fabuel, I., J. Le Douce, A. Logan, A.M. James, G. Bonvento, M.P. Murphy, A. Almeida, and J.P. Bolaños. 2016. Complex I assembly into supercomplexes determines differential mitochondrial ROS production in neurons and astrocytes. Proc. Natl. Acad. Sci. USA. 113:13063–13068. 10.1073/pnas.1613701113 27799543 PMC5135366

[bib50] Luo, Y.Z., P. He, and M.X. Qiu. 2018. FOSL1 enhances growth and metastasis of human prostate cancer cells through epithelial mesenchymal transition pathway. Eur. Rev. Med. Pharmacol. Sci. 22:8609–8615. 10.26355/eurrev_201812_16624 30575900

[bib51] Ma, Q. 2013. Role of nrf2 in oxidative stress and toxicity. Annu. Rev. Pharmacol. Toxicol. 53:401–426. 10.1146/annurev-pharmtox-011112-140320 23294312 PMC4680839

[bib52] Madan, S., B. Uttekar, S. Chowdhary, and R. Rikhy. 2022. Mitochondria lead the way: Mitochondrial dynamics and function in cellular movements in development and disease. Front. Cell Dev. Biol. 9:781933. 10.3389/fcell.2021.781933 35186947 PMC8848284

[bib53] Maib, H., and D.H. Murray. 2022. A mechanism for exocyst-mediated tethering via Arf6 and PIP5K1C-driven phosphoinositide conversion. Curr. Biol. 32:2821–2833.e6. 10.1016/j.cub.2022.04.089 35609603 PMC9382030

[bib54] Martindale, J.L., and N.J. Holbrook. 2002. Cellular response to oxidative stress: Signaling for suicide and survival. J. Cell. Physiol. 192:1–15. 10.1002/jcp.10119 12115731

[bib55] Matlashov, M.E., V.V. Belousov, and G. Enikolopov. 2014. How much H(2)O(2) is produced by recombinant D-amino acid oxidase in mammalian cells? Antioxid. Redox Signal. 20:1039–1044. 10.1089/ars.2013.5618 24020354 PMC3928830

[bib56] Mellman, D.L., M.L. Gonzales, C. Song, C.A. Barlow, P. Wang, C. Kendziorski, and R.A. Anderson. 2008. A PtdIns4,5P2-regulated nuclear poly(A) polymerase controls expression of select mRNAs. Nature. 451:1013–1017. 10.1038/nature06666 18288197

[bib57] Meng, D., Z. Li, X. Ma, L. Wu, L. Fu, and G. Qin. 2020. ETV5 overexpression contributes to tumor growth and progression of thyroid cancer through PIK3CA. Life Sci. 253:117693. 10.1016/j.lfs.2020.117693 32325133

[bib58] Obniski, R., M. Sieber, and A.C. Spradling. 2018. Dietary lipids modulate notch signaling and influence adult intestinal development and metabolism in Drosophila. Dev. Cell. 47:98–111.e5. 10.1016/j.devcel.2018.08.013 30220569 PMC6894183

[bib59] Oh, S.E., M.Y. Oh, J.Y. An, J.H. Lee, T.S. Sohn, J.M. Bae, M.G. Choi, and K.M. Kim. 2021. Prognostic value of highly expressed type VII collagen (COL7A1) in patients with gastric cancer. Pathol. Oncol. Res. 27:1609860. 10.3389/pore.2021.1609860 34512204 PMC8426344

[bib60] Osborne, S.L., C.L. Thomas, S. Gschmeissner, and G. Schiavo. 2001. Nuclear PtdIns(4,5)P2 assembles in a mitotically regulated particle involved in pre-mRNA splicing. J. Cell Sci. 114:2501–2511. 10.1242/jcs.114.13.2501 11559758

[bib61] Owusu-Ansah, E., and U. Banerjee. 2009. Reactive oxygen species prime Drosophila haematopoietic progenitors for differentiation. Nature. 461:537–541. 10.1038/nature08313 19727075 PMC4380287

[bib62] Park, Y.Y., K. Kim, S.B. Kim, B.T. Hennessy, S.M. Kim, E.S. Park, J.Y. Lim, J. Li, Y. Lu, A.M. Gonzalez-Angulo, . 2012. Reconstruction of nuclear receptor network reveals that NR2E3 is a novel upstream regulator of ESR1 in breast cancer. EMBO Mol. Med. 4:52–67. 10.1002/emmm.201100187 22174013 PMC3376834

[bib63] Poplawski, T., D. Pytel, J. Dziadek, and I. Majsterek. 2019. Interplay between redox signaling, oxidative stress, and unfolded protein response (UPR) in pathogenesis of human diseases. Oxid. Med. Cell. Longev. 2019:6949347. 10.1155/2019/6949347 31089415 PMC6476107

[bib64] Pucci, S., C. Polidoro, C. Greggi, F. Amati, E. Morini, M. Murdocca, M. Biancolella, A. Orlandi, F. Sangiuolo, and G. Novelli. 2019. Pro-oncogenic action of LOX-1 and its splice variant LOX-1Δ4 in breast cancer phenotypes. Cell Death Dis. 10:53. 10.1038/s41419-018-1279-1 30718451 PMC6362207

[bib65] Pushpa, K., S. Dagar, H. Kumar, D. Pathak, and S.V.S. Mylavarapu. 2021. The exocyst complex regulates C. elegans germline stem cell proliferation by controlling membrane Notch levels. Development. 148:dev196345. 10.1242/dev.196345 34338279

[bib66] Qi, M., Z. Liu, C. Shen, L. Wang, J. Zeng, C. Wang, C. Li, W. Fu, Y. Sun, and B. Han. 2015. Overexpression of ETV4 is associated with poor prognosis in prostate cancer: Involvement of uPA/uPAR and MMPs. Tumour Biol. 36:3565–3572. 10.1007/s13277-014-2993-7 25544710

[bib67] Ran, T., Z. Chen, L. Zhao, W. Ran, J. Fan, S. Hong, and Z. Yang. 2021. LAMB1 Is Related to the T stage and indicates poor prognosis in gastric cancer. Technol. Cancer Res. Treat. 20:15330338211004944. 10.1177/15330338211004944 33784890 PMC8020091

[bib68] Rendra, E., V. Riabov, D.M. Mossel, T. Sevastyanova, M.C. Harmsen, and J. Kzhyshkowska. 2019. Reactive oxygen species (ROS) in macrophage activation and function in diabetes. Immunobiology. 224:242–253. 10.1016/j.imbio.2018.11.010 30739804

[bib69] Rossmann, M.P., S.M. Dubois, S. Agarwal, and L.I. Zon. 2021. Mitochondrial function in development and disease. Dis. Model. Mech. 14:dmm048912. 10.1242/dmm.048912 34114603 PMC8214736

[bib70] Row, S., Y.C. Huang, and W.M. Deng. 2021. Developmental regulation of oocyte lipid intake through ‘patent’ follicular epithelium in Drosophila melanogaster. iScience. 24:102275. 10.1016/j.isci.2021.102275 33817579 PMC8005764

[bib71] Russell, O.M., G.S. Gorman, R.N. Lightowlers, and D.M. Turnbull. 2020. Mitochondrial diseases: Hope for the future. Cell. 181:168–188. 10.1016/j.cell.2020.02.051 32220313

[bib72] Saleiro, D., and L.C. Platanias. 2019. Interferon signaling in cancer. Non-canonical pathways and control of intracellular immune checkpoints. Semin. Immunol. 43:101299. 10.1016/j.smim.2019.101299 31771762 PMC8177745

[bib73] Sarmiento-Salinas, F.L., A. Delgado-Magallón, J.B. Montes-Alvarado, D. Ramírez-Ramírez, J.C. Flores-Alonso, P. Cortés-Hernández, J. Reyes-Leyva, I. Herrera-Camacho, M. Anaya-Ruiz, R. Pelayo, . 2019. Breast cancer subtypes present a differential production of reactive oxygen species (ROS) and susceptibility to antioxidant treatment. Front. Oncol. 9:480. 10.3389/fonc.2019.00480 31231612 PMC6568240

[bib74] Schieber, M., and N.S. Chandel. 2014. ROS function in redox signaling and oxidative stress. Curr. Biol. 24:R453–R462. 10.1016/j.cub.2014.03.034 24845678 PMC4055301

[bib75] Sethuraman, A., M. Brown, R. Krutilina, Z.H. Wu, T.N. Seagroves, L.M. Pfeffer, and M. Fan. 2018. BHLHE40 confers a pro-survival and pro-metastatic phenotype to breast cancer cells by modulating HBEGF secretion. Breast Cancer Res. 20:117. 10.1186/s13058-018-1046-3 30285805 PMC6167787

[bib76] Shcherbata, H.R., C. Althauser, S.D. Findley, and H. Ruohola-Baker. 2004. The mitotic-to-endocycle switch in Drosophila follicle cells is executed by Notch-dependent regulation of G1/S, G2/M and M/G1 cell-cycle transitions. Development. 131:3169–3181. 10.1242/dev.01172 15175253

[bib77] Shen, Y., X. Wang, J. Xu, and L. Lu. 2017. SerpinE2, a poor biomarker of endometrial cancer, promotes the proliferation and mobility of EC cells. Cancer Biomark. 19:271–278. 10.3233/CBM-160442 28453461 PMC13020735

[bib78] Shimokawa, H. 2020. Reactive oxygen species in cardiovascular health and disease: Special references to nitric oxide, hydrogen peroxide, and rho-kinase. J. Clin. Biochem. Nutr. 66:83–91. 10.3164/jcbn.19-119 32231403 PMC7093293

[bib79] Sieber, M.H., M.B. Thomsen, and A.C. Spradling. 2016. Electron transport chain remodeling by GSK3 during oogenesis connects nutrient state to reproduction. Cell. 164:420–432. 10.1016/j.cell.2015.12.020 26824655 PMC6894174

[bib80] Sies, H., C. Berndt, and D.P. Jones. 2017. Oxidative stress. Annu. Rev. Biochem. 86:715–748. 10.1146/annurev-biochem-061516-045037 28441057

[bib81] Sies, H., and D.P. Jones. 2020. Reactive oxygen species (ROS) as pleiotropic physiological signalling agents. Nat. Rev. Mol. Cell Biol. 21:363–383. 10.1038/s41580-020-0230-3 32231263

[bib82] Sinenko, S.A., J. Shim, and U. Banerjee. 2011. Oxidative stress in the haematopoietic niche regulates the cellular immune response in Drosophila. EMBO Rep. 13:83–89. 10.1038/embor.2011.223 22134547 PMC3246251

[bib83] Steinhaeuser, S.S., E. Morera, Z. Budkova, A. Schepsky, Q. Wang, O. Rolfsson, A. Riedel, A. Krueger, B. Hilmarsdottir, G.M. Maelandsmo, . 2020. ECM1 secreted by HER2-overexpressing breast cancer cells promotes formation of a vascular niche accelerating cancer cell migration and invasion. Lab. Invest. 100:928–944. 10.1038/s41374-020-0415-6 32203150

[bib84] Sullivan, L.B., and N.S. Chandel. 2014. Mitochondrial reactive oxygen species and cancer. Cancer Metab. 2:17. 10.1186/2049-3002-2-17 25671107 PMC4323058

[bib85] Sun, C., S. Ma, Y. Chen, N.H. Kim, S. Kailas, Y. Wang, W. Gu, Y. Chen, J.P.W. Tuason, C. Bhan, . 2022. Diagnostic value, prognostic value, and immune infiltration of LOX family members in liver cancer: Bioinformatic analysis. Front. Oncol. 12:843880. 10.3389/fonc.2022.843880 35311155 PMC8931681

[bib86] Sun, J., L. Smith, A. Armento, and W.M. Deng. 2008. Regulation of the endocycle/gene amplification switch by Notch and ecdysone signaling. J. Cell Biol. 182:885–896. 10.1083/jcb.200802084 18779369 PMC2528591

[bib87] Synek, L., R. Pleskot, J. Sekereš, N. Serrano, N. Vukašinović, J. Ortmannová, M. Klejchová, P. Pejchar, K. Batystová, M. Gutkowska, . 2021. Plasma membrane phospholipid signature recruits the plant exocyst complex via the EXO70A1 subunit. Proc. Natl. Acad. Sci. USA. 118:e2105287118. 10.1073/pnas.2105287118 34470819 PMC8433549

[bib88] Tait, S.W., and D.R. Green. 2012. Mitochondria and cell signalling. J. Cell Sci. 125:807–815. 10.1242/jcs.099234 22448037 PMC3311926

[bib89] Tamma, G., G. Valenti, E. Grossini, S. Donnini, A. Marino, R.A. Marinelli, and G. Calamita. 2018. Aquaporin membrane channels in oxidative stress, cell signaling, and aging: Recent advances and research trends. Oxid. Med. Cell. Longev. 2018:1501847. 10.1155/2018/1501847 29770164 PMC5892239

[bib90] Tanaka, H., I. Matsumura, S. Ezoe, Y. Satoh, T. Sakamaki, C. Albanese, T. Machii, R.G. Pestell, and Y. Kanakura. 2002. E2F1 and c-Myc potentiate apoptosis through inhibition of NF-kappaB activity that facilitates MnSOD-mediated ROS elimination. Mol. Cell. 9:1017–1029. 10.1016/S1097-2765(02)00522-1 12049738

[bib91] Tao, Y., N. Gross, X. Fan, J. Yang, M. Teng, X. Li, G. Li, Y. Zhang, and Z. Huang. 2018. Identification of novel enriched recurrent chimeric COL7A1-UCN2 in human laryngeal cancer samples using deep sequencing. BMC Cancer. 18:248. 10.1186/s12885-018-4161-8 29499655 PMC5834868

[bib92] Tatsuta, T., K. Model, and T. Langer. 2005. Formation of membrane-bound ring complexes by prohibitins in mitochondria. Mol. Biol. Cell. 16:248–259. 10.1091/mbc.e04-09-0807 15525670 PMC539169

[bib93] Teixeira, F.K., C.G. Sanchez, T.R. Hurd, J.R. Seifert, B. Czech, J.B. Preall, G.J. Hannon, and R. Lehmann. 2015. ATP synthase promotes germ cell differentiation independent of oxidative phosphorylation. Nat. Cell Biol. 17:689–696. 10.1038/ncb3165 25915123 PMC4573567

[bib94] Thapa, N., Y. Sun, M. Schramp, S. Choi, K. Ling, and R.A. Anderson. 2012. Phosphoinositide signaling regulates the exocyst complex and polarized integrin trafficking in directionally migrating cells. Dev. Cell. 22:116–130. 10.1016/j.devcel.2011.10.030 22264730 PMC3266520

[bib95] Vyas, S., E. Zaganjor, and M.C. Haigis. 2016. Mitochondria and cancer. Cell. 166:555–566. 10.1016/j.cell.2016.07.002 27471965 PMC5036969

[bib96] Wan, X., S. Guan, Y. Hou, Y. Qin, H. Zeng, L. Yang, Y. Qiao, S. Liu, Q. Li, T. Jin, . 2021. FOSL2 promotes VEGF-independent angiogenesis by transcriptionnally activating Wnt5a in breast cancer-associated fibroblasts. Theranostics. 11:4975–4991. 10.7150/thno.55074 33754039 PMC7978317

[bib97] Wang, N., Y. Xu, Q. Guo, C. Zhu, W. Zhao, W. Qian, and M. Zheng. 2021. Effects of miR-132-3p on progress and epithelial mesenchymal transition of non-small cell lung cancer via regulating KLF7. J. Thorac. Dis. 13:2426–2436. 10.21037/jtd-21-353 34012590 PMC8107552

[bib98] Wang, Q., Y. Shi, H.J. Butler, J. Xue, G. Wang, P. Duan, and H. Zheng. 2017. Role of delta-like ligand-4 in chemoresistance against docetaxel in MCF-7 cells. Hum. Exp. Toxicol. 36:328–338. 10.1177/0960327116650006 27334972

[bib99] Wang, W., F. Zhao, X. Ma, G. Perry, and X. Zhu. 2020. Mitochondria dysfunction in the pathogenesis of Alzheimer’s disease: Recent advances. Mol. Neurodegener. 15:30. 10.1186/s13024-020-00376-6 32471464 PMC7257174

[bib100] Wang, Z., Q. Zhou, A. Li, W. Huang, Z. Cai, and W. Chen. 2019. Extracellular matrix protein 1 (ECM1) is associated with carcinogenesis potential of human bladder cancer. OncoTargets Ther. 12:1423–1432. 10.2147/OTT.S191321 PMC6389008 30863109

[bib102] Ward, E.J., X. Zhou, L.M. Riddiford, C.A. Berg, and H. Ruohola-Baker. 2006. Border of Notch activity establishes a boundary between the two dorsal appendage tube cell types. Dev. Biol. 297:461–470. 10.1016/j.ydbio.2006.05.021 16828735

[bib103] Widjaja, A.A., S. Viswanathan, J.G. Wei Ting, J. Tan, S.G. Shekeran, D. Carling, W.W. Lim, and S.A. Cook. 2022. IL11 stimulates ERK/P90RSK to inhibit LKB1/AMPK and activate mTOR initiating a mesenchymal program in stromal, epithelial, and cancer cells. iScience. 25:104806. 10.1016/j.isci.2022.104806 35992082 PMC9386112

[bib104] Yen, C.Y., C.H. Chen, C.H. Chang, H.F. Tseng, S.Y. Liu, L.Y. Chuang, C.H. Wen, and H.W. Chang. 2009. Matrix metalloproteinases (MMP) 1 and MMP10 but not MMP12 are potential oral cancer markers. Biomarkers. 14:244–249. 10.1080/13547500902829375 19489686

[bib105] Yoneda, M., Y.S. Hirokawa, A. Ohashi, K. Uchida, D. Kami, M. Watanabe, T. Yokoi, T. Shiraishi, and S. Wakusawa. 2010. RhoB enhances migration and MMP1 expression of prostate cancer DU145. Exp. Mol. Pathol. 88:90–95. 10.1016/j.yexmp.2009.09.010 19782069

[bib106] Yuan, Z.Y., T. Dai, S.S. Wang, R.J. Peng, X.H. Li, T. Qin, L.B. Song, and X. Wang. 2014. Overexpression of ETV4 protein in triple-negative breast cancer is associated with a higher risk of distant metastasis. OncoTargets Ther. 7:1733–1742. 10.2147/OTT.S66692 PMC4196788 25328406

[bib107] Zhang, D., Y. Dai, Y. Cai, T. Suo, H. Liu, Y. Wang, Z. Cheng, and H. Liu. 2016. KLF2 is downregulated in pancreatic ductal adenocarcinoma and inhibits the growth and migration of cancer cells. Tumour Biol. 37:3425–3431. 10.1007/s13277-015-4053-3 26449825

[bib108] Zhang, J., Q. Wu, L. Zhu, S. Xie, L. Tu, Y. Yang, K. Wu, Y. Zhao, Y. Wang, Y. Xu, . 2022a. SERPINE2/PN-1 regulates the DNA damage response and radioresistance by activating ATM in lung cancer. Cancer Lett. 524:268–283. 10.1016/j.canlet.2021.10.001 34648881

[bib109] Zhang, W., X. Huang, R. Huang, H. Zhu, P. Ye, X. Lin, S. Zhang, M. Wu, and F. Jiang. 2022b. MMP1 Overexpression promotes cancer progression and associates with poor outcome in head and neck carcinoma. Comput. Math. Methods Med. 2022:3058342. 10.1155/2022/3058342 36105241 PMC9467809

[bib110] Zhang, Y., S. Choksi, K. Chen, Y. Pobezinskaya, I. Linnoila, and Z.G. Liu. 2013. ROS play a critical role in the differentiation of alternatively activated macrophages and the occurrence of tumor-associated macrophages. Cell Res. 23:898–914. 10.1038/cr.2013.75 23752925 PMC3698641

[bib111] Zhao, K., W. Wang, O.J. Rando, Y. Xue, K. Swiderek, A. Kuo, and G.R. Crabtree. 1998. Rapid and phosphoinositol-dependent binding of the SWI/SNF-like BAF complex to chromatin after T lymphocyte receptor signaling. Cell. 95:625–636. 10.1016/S0092-8674(00)81633-5 9845365

[bib112] Zhou, B., and R. Tian. 2018. Mitochondrial dysfunction in pathophysiology of heart failure. J. Clin. Invest. 128:3716–3726. 10.1172/JCI120849 30124471 PMC6118589

[bib113] Zhou, D., L. Shao, and D.R. Spitz. 2014. Reactive oxygen species in normal and tumor stem cells. Adv. Cancer Res. 122:1–67. 10.1016/B978-0-12-420117-0.00001-3 24974178 PMC4207279

[bib114] Zhu, Z., J. Song, Y. Guo, Z. Huang, X. Chen, X. Dang, Y. Huang, Y. Wang, W. Ou, Y. Yang, . 2020. LAMB3 promotes tumour progression through the AKT-FOXO3/4 axis and is transcriptionally regulated by the BRD2/acetylated ELK4 complex in colorectal cancer. Oncogene. 39:4666–4680. 10.1038/s41388-020-1321-5 32398865

[bib115] Zong, W.X., J.D. Rabinowitz, and E. White. 2016. Mitochondria and cancer. Mol. Cell. 61:667–676. 10.1016/j.molcel.2016.02.011 26942671 PMC4779192

[bib116] Zou, R., J. Tao, J. Qiu, W. Shi, M. Zou, W. Chen, W. Li, N. Zhou, S. Wang, L. Ma, and X. Chen. 2021. Ndufs1 deficiency aggravates the mitochondrial membrane potential dysfunction in pressure overload-induced myocardial hypertrophy. Oxid. Med. Cell. Longev. 2021:5545261. 10.1155/2021/5545261 33763166 PMC7952157

