# Supplementary figures and images for: Non-cell autonomous regulation of cell–cell signaling and differentiation by mitochondrial ROS
Source: J Cell Biol. 2024 Nov 13;223(12):e202401084. doi: 10.1083/jcb.202401084 (PMC11561560; doi:10.1083/jcb.202401084)

Figure 4 G

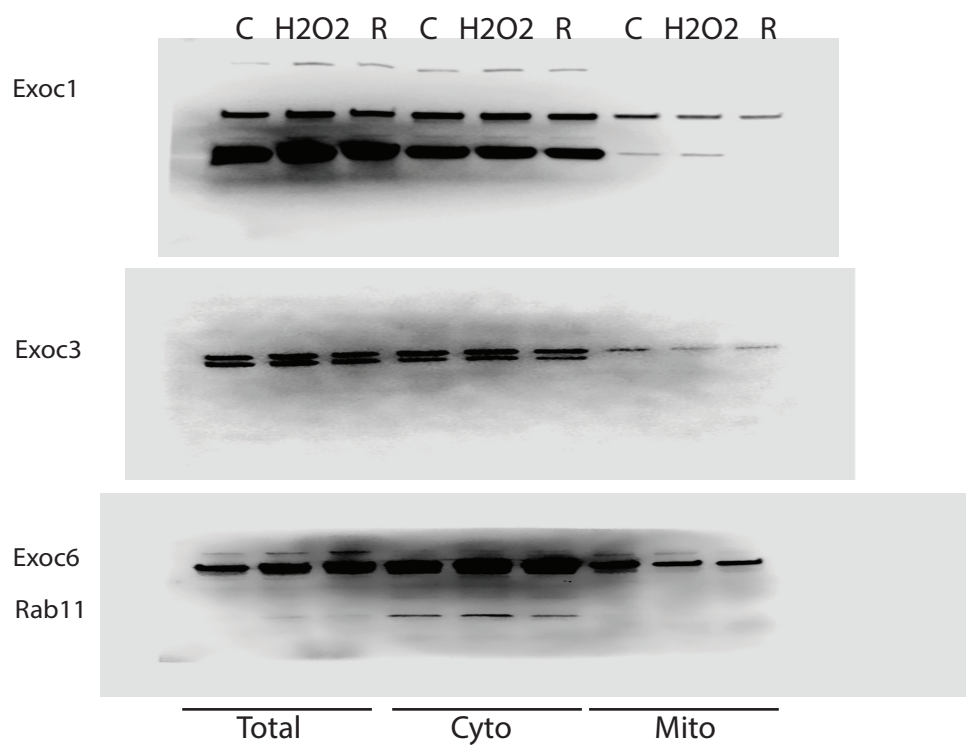

Figure 4 H

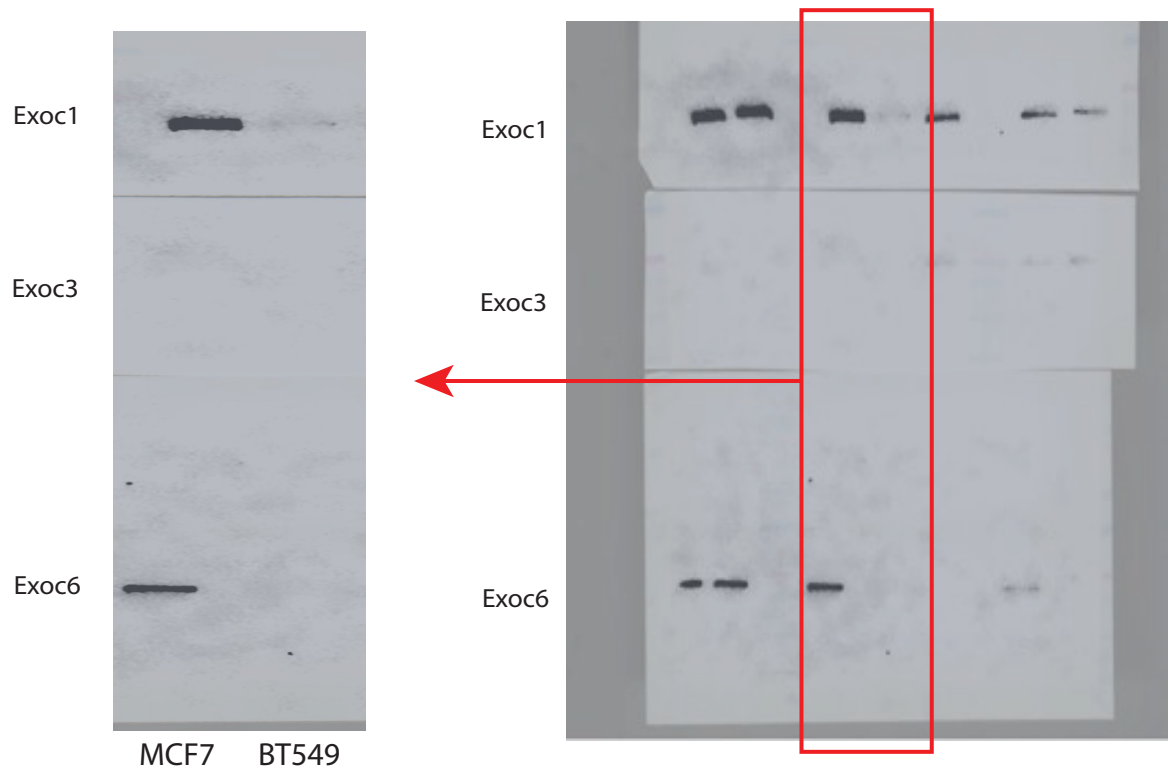

Figure 4 I

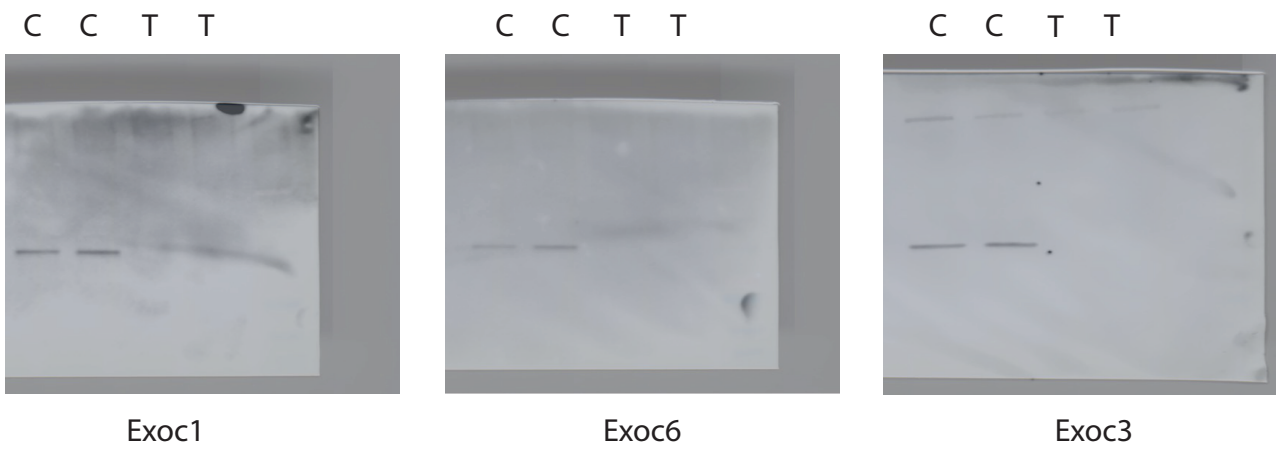

Supplement: SourceData F4 — is the source file for Fig. 4. [file JCB_202401084_SourceDataF4.pdf]
